# Investigation of *Armigeres subalbatus*, a vector of zoonotic *Brugia pahangi* filariasis in plantation areas in Suratthani, Southern Thailand

**DOI:** 10.1016/j.onehlt.2021.100261

**Published:** 2021-04-30

**Authors:** Apiradee Intarapuk, Adisak Bhumiratana

**Affiliations:** a Faculty of Veterinary Medicine, Mahanakorn University of Technology, Bangkok 10530, Thailand; b Faculty of Public Health, Thammasat University, Rangsit Campus, Pathumthani 12121, Thailand

**Keywords:** *Armigeres subalbatus*, *Brugia pahangi*, Filarial *β-tubulin* genes, Local landscape variation, Plantation ecotype, Touchup-nested PCR

## Abstract

In recent years, children in Thailand have been infected with zoonotic *Brugia pahangi.* However, the local environment of rubber or oil palm plantations, which would increase their exposure to risk factors of the infection due to mosquito transmission, is unclear. The present study first sought to determine the extent to which variations in the local landscape, such as the elevated versus low-lying ecotope of rubber or oil palm plantations, in a 2-km radius of the geographically defined landscape in a rural area of Suratthani, Southern Thailand could influence the abundance of *Armigeres subalbatus* and its susceptibility to zoonotic filarial parasite infections compared to *Mansonia*, *Aedes*, and *Culex*, and *Coquillettidia*. Thereafter, the filarial larvae found in the infected mosquitoes were molecularly investigated. *Ar. subalbatus* plantation ecotype was not only found to outnumber the local mosquitoes, but was identified as the predominant species that adapted well to the elevated ecotopes of the rubber or oil palm plantations, which existed at altitudes of 60–80 m. The overall rate of zoonotic filarial parasite infections (L_1_, L_2_, or L_3_ larvae) of *Ar. subalbatus* was 2.5% (95% CI, −0.2 to 4.1), with an average L_3_ load of 2.3 larvae per infected *Ar. subalbatus* (95% CI, −0.6 to 13.0); this is because the infections were found to be concentrated in the elevated ecotopes alone. Based on filarial orthologous *β-tubulin* gene-specific touchup-nested PCR and sequence analysis using 30 L_3_ larva clones as representatives of 9 *Ar. subalbatus* infectious pools, *Ar. subalbatus* either carried *B. pahangi* or *Dirofilaria immitis*, or both species. Such findings suggest that *Ar. subalbatus* might have played an imperative role in the transmission of *B. pahangi* in the plantation areas infested with *Ar. subalbatus*.

## Highlights

• *Ar. subalbatus* is the natural vector for zoonotic *Brugia pahangi*.
• *Ar. subalbatus* plantation ecotype adapted to rubber or oil palm plantations.
• Local landscape variation influencing its abundance and susceptibility to zoonotic filarial infection.
• Touchup-nested PCRs specific for amplifying filarial beta-tubulin genes of L_3_ larvae present in *Ar. subalbatus*.
• Implying that *Ar. subalbatus* carried either *B. pahangi* or *D. immitis*, or both.

## Graphical abstract

**Figure:**
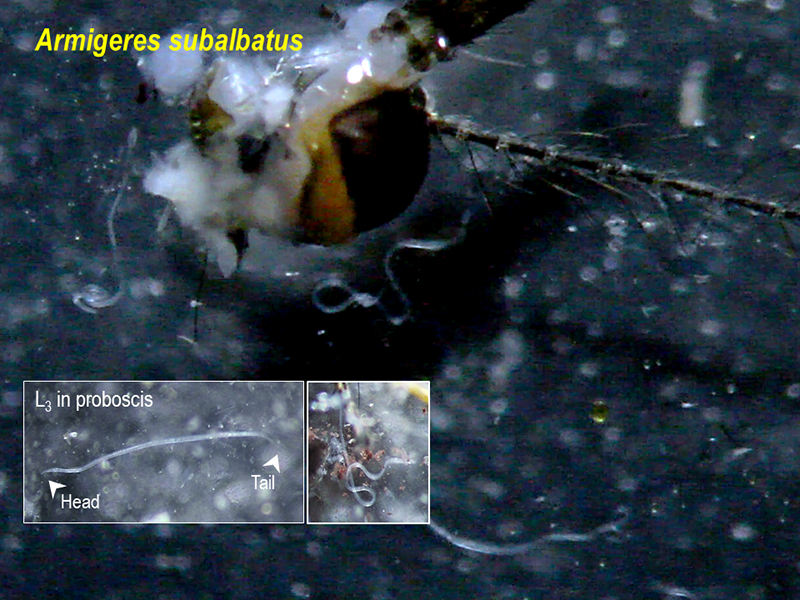

## 1 Introduction

Vector-borne parasitic zoonosis is geographically distributed on a global scale [1,2]. As a result, it is a public health problem that has a disproportionate burden due to its occurrence among persons that live below the poverty level [[3], [4], [5]]. Re-emerging and emerging zoonotic *Brugia malayi* and *Brugia pahangi* lymphatic filarial parasites can be transmitted by domestic animal reservoirs, such as cats and dogs, to humans [[6], [7], [8], [9]]. Of note, emerging zoonotic infections with *B. pahangi* parasites have recently been reported to be sporadic in Southeast Asia (SEA) [7,8]. Owing to expansion in geographical locations, host or vector range, and the dynamics of transmission patterns that involve biological, ecological, and social factors in different complex eco-epidemiological settings [5,10], the prevalence of lingering zoonotic infections with *B. pahangi* parasites is unknown.

In recent years, there has been an emergence of zoonotic *B. pahangi* infections in children younger than 2 years of age in Thailand (see the supplementary Table S1). Thus, addressing the risk at the interface of human, animal, and the environment, and understanding the vulnerability of children and how they acquire these infections locally through mosquito transmission, are urgently required. Children might be vulnerable to infection with *B. pahangi* owing to poor social or environmental conditions [3]; the infestation of local mosquitoes from five genera, namely *Armigeres*, *Anopheles*, *Mansonia*, *Aedes*, and *Culex* [11]; and the presence of cats or dogs infected with *B. pahangi* [12] within a 2-km radius of their houses. Of the five mosquito genera mentioned above, *Mansonia* mosquitoes, such as *Ma. uniformis*, *Ma. indiana*, *Ma. annulifera*, and *Ma. bonneae*, are the main vectors for *B. malayi* in Thailand [13] and other countries in SEA [14]. *Armigeres subalbatus* is the natural vector for zoonotic *B. pahangi* [15,16] and may also transmit *B. pahangi* to humans in SEA [8]. There are two ecotypes of *Ar. subalbatus* that share common characteristics: the forest and plantation ecotypes. The forest ecotype is indigenous to the forest and forest fringe and can breed in natural containers with organic substances. In contrast, the plantation ecotype is well adapted to plantation areas where breeding can occur in either artificial or natural containers with organic substances. The present study attempted to clarify some ecologic and epidemiologic questions regarding the occurrence of zoonotic *B. pahangi* infections in children by determining the fitness of *Ar. subalbatus* plantation ecotype (whether this species adapted to the local environment of rubber or oil palm plantations) and its potential to transmit epizootic *B. pahangi* parasites.

We mimicked local environmental conditions of cases infected with *B. pahangi* in the plantation areas of Suratthani, Southern Thailand (case no. 1), and Rayong, Eastern Thailand (case no. 2) (Table S1) by employing a 2-km radius of geographically defined local landscape as the study area. This study area covered four different ecotopes, which served as the study sites in Suratthani. Thereafter, we sought to empirically determine the extent to which variations in the local landscape could influence the infestation of *Ar. subalbatus* plantation ecotypes and zoonotic filarial infections (with L_1_/L_2_/L_3_ juveniles) of *Ar. subalbatus* compared to other local mosquitoes; and to investigate the representative L_3_ larva clones of *B. pahangi,* originally derived from any infectious mosquito pool using a newly developed touchup-nested PCR specific for the amplification of filarial orthologous *β-tubulin* genes and sequence analysis [[17], [18], [19]].

## 2 Materials and methods

### 2.1 Study area and site

Prior to the study carried out in 2013, the local environmental conditions of the receptive plantation areas (Table S1) were considered ideal for the establishment of the selection criterion (i.e., the selection of the local landscape as the study area where domestic animal reservoirs carrying *B. pahangi* and human blood-seeking mosquitoes inhabit). The selection criteria were based on field data obtained using field surveys. A topographical survey was conducted to gather spatial information and to locate both natural and man-made land surface features using land use maps with elevation contours and geographical positioning system (GPS). This provided the correct information of the scaled and detailed boundary of the selected study area and the GPS coordinates of the ecotopes, which were the selected study sites, based on spatial considerations such as latitude, longitude, altitude, rubber or oil palm plantation polygons, roads, water bodies, building, built-up land, and potential larval breeding sites [20,21]. The ecotope can be defined as a small-scale landscape of the plantation area that is geographically associated with the infestation of locally adapted mosquitoes (e.g., *Mansonia*, *Armigeres*, *Aedes*, *Culex*, *Coquillettidia*, or *Anopheles*). Parasitological survey was carried out to examine zoonotic filarial infections in domestic cats or dogs within the boundary of the selected study area. Blood samples collected from cats or dogs at night were used for this survey. Larval survey was carried out to assess the availability of larval habitats of local mosquitoes within, or proximal to, the selected ecotopes. Household survey was conducted to assess the physical environmental conditions inside and outside houses with domestic animals.

**Fig. 1:**
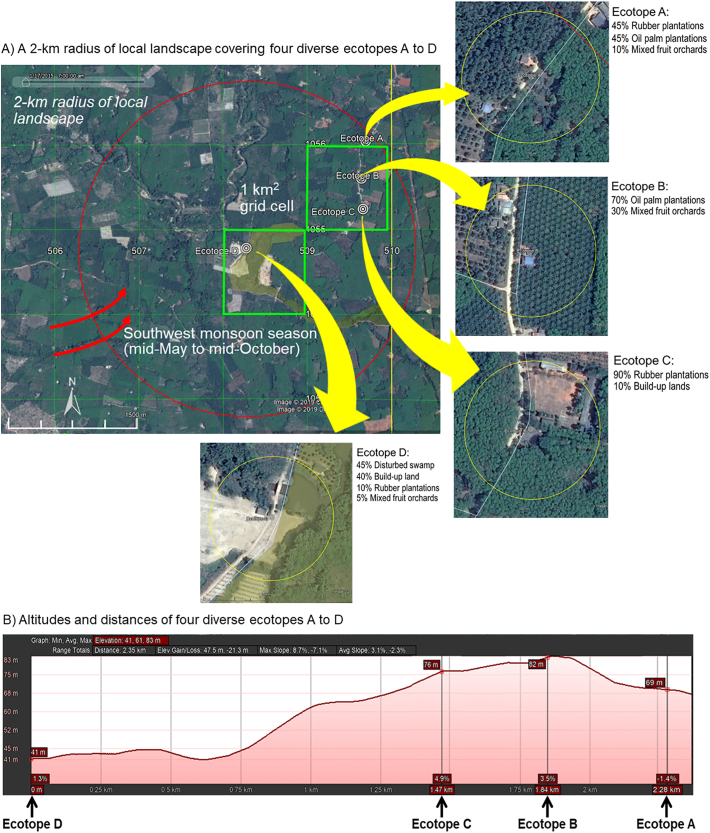
Local landscape of the study area and study sites. A) Google Earth maps illustrating a 2-km radius with variations in the geographically defined local landscape and four diverse ecotopes (A to D). A 100-m radius of land use is shown. B) Location of the four ecotopes with different altitudes and distances. Google Earth maps illustrating diverse filariasis ecotopes (A to D). Ecotopes A to C (A) represent land use and land cover distinguishable from ecotope D and (B) geographically associated with the infestation of *Mansonia* vectors.

The study area that met the selection criteria covered a 2-km radius of geographically defined local landscape. The study area was also comprised of four diverse ecotopes, which served as the study sites using a 1-km^2^ universal transverse mercator (UTM) grid cell (Fig. 1A). Of note, the study area was geographically associated with the infestation of local mosquitoes (i.e., *Mansonia*, *Armigeres*, *Aedes*, *Culex*, and *Coquillettidia*). The elevated ecotopes (i.e., A to C) that were mainly covered with either rubber or oil palm plantations were located approximately 70 m above sea level (MASL), whereas the low-lying ecotope (i.e., D) with only 10% rubber plantation was located at 40 MASL in a disturbed swamp, which is the breeding habitat of *Mansonia* mosquitoes (Fig. 1B). The locations of ecotopes A to D were used as the sites of sampling for local mosquitoes as shown in Table S2.

### 2.2 Mosquito collection and dissection

Periodic assessments of species compositions and abundances of the local mosquito populations were carried out between May 2014 and May 2015. At each timepoint of mosquito collection from each ecotope, the human landing catch (HLC) collection method was used to detect the species and numbers of mosquitoes, both indoors and outdoors, between 18:00 h and 21:00 h for 3 consecutive nights (i.e., 3 pools of identified mosquitoes were obtained). The taxonomic identification of individual adult female mosquitoes was blindly performed by two expert entomologists. The abundance of local mosquitoes was expressed as man landing rate (MLR), which infers the number of human blood-seeking mosquitoes per night per person for each ecotope.

**Fig. 2:**
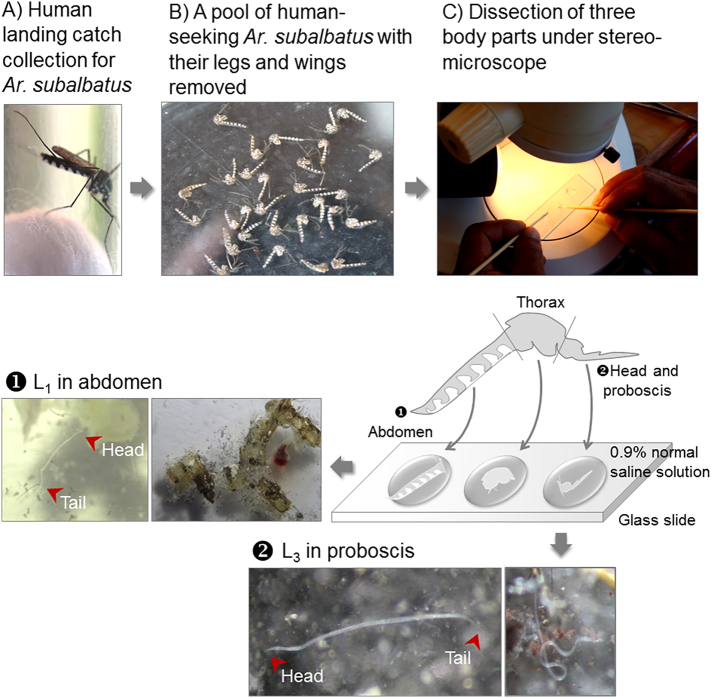
*Ar. subalbatus* infectious mosquito pool. Based on HLC collection (A), a pool no. of BAP1, including 29 *Ar. subalbatus* adult female mosquitoes obtained from ecotope B, was used to represent the *Ar. subalbatus* infectious mosquito pool that was individually dissected into three body parts (B—C). Compared to L_1_ obtained from the abdomen ①, the isolation of single L_3_ larva clone obtained from the proboscis ② is shown.

All identified adult female mosquitoes were individually examined for the presence of L_1_, L_2_, or L_3_ larval stages by dissection under a stereomicroscope (Fig. 2). None of the mosquito pools of *Culex*, *Aedes*, *Mansonia*, and *Coquillettidia* collected from the studied ecotopes was positive, except for the infectious pools of *Ar. subalbatus* (Fig. 2). The number of larvae recovered from the abdomen (L_1_), thorax (L_2_), and head and proboscis (L_3_) was tallied [18], and the average number and range of L_3_ per infected mosquito were presented. The infectious mosquito pool must have had at least one adult female mosquito infected with any of the L_1_, L_2_, or L_3_ larval stages. For example, the abundance of the *Ar. subalbatus* infectious pool was expressed as infectious MLR, which infers the infectious number of human blood-seeking *Ar. subalbatus* per night per person for each ecotope. The infection rate (%) for each ecotope was expressed as the number of *Ar. subalbatus* adult female mosquitoes infected with any larval stages (L_1_, L_2_, or L_3_) in the total number of *Ar. subalbatus* adult female mosquitoes collected by HLC, multiplied by 100. The 95% confidence interval (CI) was used to estimate the infection rate for *Ar. subalbatus* in all ecotopes and the L_3_ load for the *Ar. subalbatus* infectious pools.

### 2.3 Genomic DNA preparation of L_3_ and positive controls

Immediately after mosquito dissection (Fig. 2), single L_3_ larva clones originally obtained from the 9 *Ar. subalbatus* infectious pools of ecotopes A to C were isolated under carefully controlled conditions in the field. Of the 56 L_3_ larva clones obtained, 30 were used as representatives of the L_3_ gDNA templates: AAP1 (L01 to L12 clones), AAP2 (L13 to L15 clones), AAP3 (L16 to L18 clones), BAP1 (L19 to L22 clones), BAP2 (L23 clone), BAP3 (L24 to L25 clones), CAP1 (L26 clone), CAP2 (L27 clone), and CAP3 (L28 to L30 clones). These clones were separately prepared for gDNA extraction using the QIAamp® Blood Mini Kit (QIAGEN, Germany), as described elsewhere [19], with modifications. Finally, the eluted L_3_ gDNA solution (approximate 100–200 μl), which had a 260/280 absorbance ratio of 1.8–2.0, contained the average gDNA content per L_3_ clone of 994.6 ng, or 7.4 to 12.5 ng/μl.

The positive controls included purified gDNA templates of microfilaremic (Mf) bloods harboring a wide range of microfilarial densities (Mf/ml): *Wuchereria bancrofti* MMO7 (1246), MDA1 (252), and MMO6 (13) patient isolates [19]; *Brugia malayi* NT01 (673), NT02 (166), and NT08 (367) patient isolates; *B. pahangi* DA08 (167) dog isolate and CA12 (300) cat isolate; and *Dirofilaria immitis* Di106 (>5000) and Di101 (2633) dog isolates. All purified Mf gDNA samples were prepared using the QIAamp® Blood Mini Kit (QIAGEN, Germany) according to the methods described elsewhere [17,19]. Negative controls, such as human, cat, dog, or mosquito gDNAs and nuclease-free deionized water, were used throughout the study.

### 2.4 Amplification of orthologous *β-tubulin* genes of L_3_

#### 2.4.1 Primer design

**Fig. 3:**
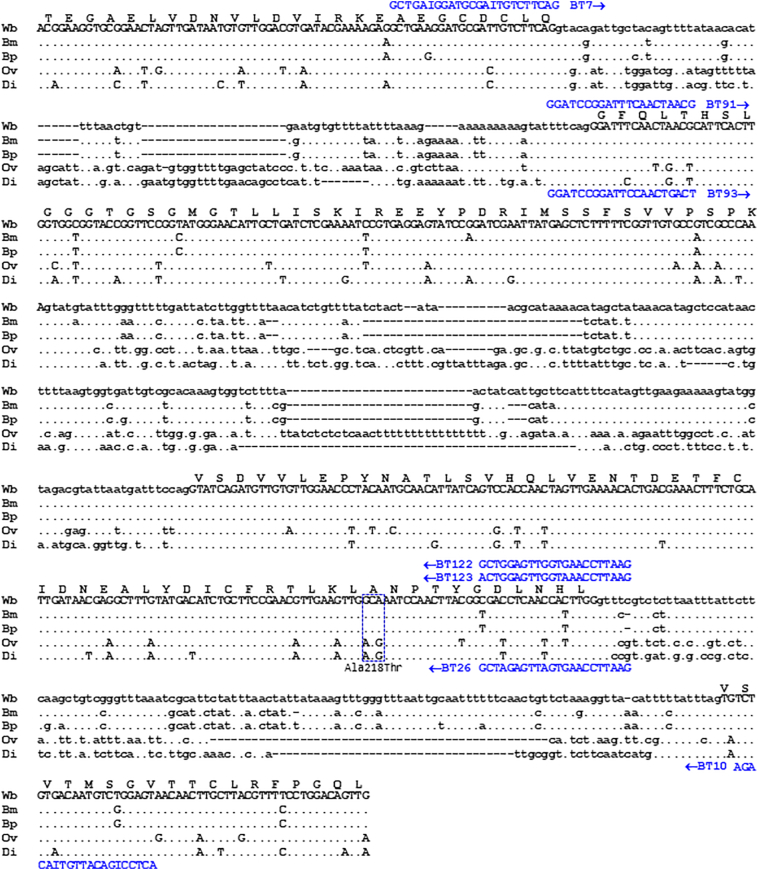
Multiple sequence alignment of partially homologous *β-tubulin* genes of human and non-human filariids, which are involved in benzimidazole susceptibility. The retrieved nucleotide sequences (accession no. and positions): *Brugia malayi* (Bm) (BRQD553TR, 3–789), *Brugia pahangi* (Bp) (M36380, 2267–3054), *Wuchereria bancrofti* (Wb) (AY705383, 109–916), *Onchocerca volvulus* (Ov) (AF019886, 1582–2400), and *Dirofilaria immitis* (Di) (HM596854, 1462–2244) are shown as coding (upper case) and non-coding (lower case) sequences. The gap (insertion/deletion) was generated to maximize the homology representing conserved (•) and degenerate nucleotide residues. The deduced amino acid sequences (Thr107 to Leu234) of conserved β-tubulin homologs of all taxa are shown along with the amino acid substitutions (blue color-dashed boxes). The primers that specifically annealed to the regions of target L_3_ gDNAs of *B. malayi* (Bm*tubb*), *B. pahangi* (Bp*tubb*), *D. immitis* (Di*tubb*), and *W. bancrofti* (Wb*tubb*), and their direction of amplification (arrows) are shown. (For interpretation of the references to color in this figure legend, the reader is referred to the web version of this article.)

The nucleotide sequences of filarial orthologous *β-tubulin* genes were retrieved from the genome databases, GenBank, EMBL, DDBJ, and DFCI. Their homology was analyzed using multiple sequence alignment (Fig. 3) according to the methods described elsewhere [17,19]. Based on the conserved sequences retained in the filarial *β-tubulin* orthologs, the primer sets designed for the touchup-nested PCR formats (Fig. 3 and Table 1) to yield authentically amplified L_3_ DNA fragments were tested for specificity and their physical properties, according to the methods described elsewhere [17,19].

**Table 1:** Primers used in touchup-nested PCRs specific for the orthologous *β-tubulin* genes using L_3_ gDNAs.

Primer name	Direction/Sequence (5′ to 3′)^d^	Target DNA: amplicons with expected size (bp)	Reference
Heterologous primers^a^			
BT7	Forward/GCTGAIGGATGCGAITGTCTTCAG	Wb*tubb* (726),	This study
BT10	Reverse/ACTCCIGACATTGTIACAGA	Di*tubb* (701),	
		Bm*tubb* (705),	
		Bp*tubb* (706)	
Species-specific primers^b^			
BT91	Forward/GGATCCGGATTTCAACTAACG	Wb*tubb* (493)	[16]
BT122	Reverse/GAATTCCAAGTGGTTGAGGTCG		
BT93	Forward/GGATCCGGATTCCAACTGACT	Di*tubb* (477)	This study
BT26	Reverse/GAATTCCAAGTGATTGAGATCG		
*Brugia*-specific primers^c^			
BT91	Forward/GGATCCGGATTTCAACTAACG	Bm*tubb* (468),	This study
BT123	Reverse/GAATTCCAAATGGTTGAGGTCA	Bp*tubb* (468)	

Primer sets used in touchup-nested PCRs: ^a^first-round amplification of orthologous *β-tubulin* genes and ^b,c^second-round amplification of *Brugia* (Bm*tubb* or Bp*tubb*), *D. immitis* (Di*tubb*), and *W. bancrofti* (Wb*tubb*) in separate reactions.

^d^Underlined sequences of primers represent the 5′ modification sites with additional recognition sequences, *Bam*H I (GGATCC) and *Eco*R I (GAATTC), while ^a^the degenerated inosine (I) can substitute any base changed (A, G, T, or C).

#### 2.4.2 Touchup-nested PCR

Initially, the optimization of the touchup-nested PCR (TUPCR) program was carried out empirically using a bracket of specific primer-template annealing temperatures in a 25-μl reaction volume, as described elsewhere [19], except that 5 μl (approximately 30–60 ng) of purified L_3_ gDNA template was used. In primary TUPCR, the heterologous primers, BT7 and BT10, that could specifically amplify 701 to 726 bp amplicons were originally derived from orthologous *β-tubulin* genes of human or non-human filarial parasites. The reaction was performed with an initial denaturation at 95 °C for 4 min, followed by 35 cycles of denaturation at 95 °C for 1 min, annealing at 57 °C for 1 min and polymerization at 72 °C for 1 min. The final extension was performed at 72 °C for 4 min.

For the secondary TUPCRs containing species-specific or *Brugia*-specific primer sets, the PCR ingredients were similar to those of the first reaction, except that 2 μl of the primary TUPCR products was used as a template. A touchup program (or TU5660) was performed with initial denaturation at 95 °C for 1 min, followed by 5 cycles with successive annealing temperature increments of 1 °C in every cycle. For the first 5 cycles, the reaction mixture was heated at 95 °C for 1 min, followed by annealing at 56 °C → 60 °C for 1 min and polymerization at 72 °C for 1 min. The subsequent 30 cycles of amplification were similar, except that the annealing temperature was 60 °C for 1 min. Lastly, the extension was performed at 72 °C for 4 min. TUPCRs containing both positive controls (approximately 20–30 ng of Mf gDNA templates) and negative controls were performed in the same manner as those containing the L_3_ gDNA templates.

### 2.5 Post-PCR analysis and sequencing

Electrophoresis of the PCR products with expected sizes and the determination of the homology of all sequenced amplicons at the DNA and protein levels were accomplished using the methods described elsewhere [17,19]. The nucleotide sequences from this study were deposited in the GenBank database (accession numbers): *W. bancrofti* (MT674270-MT674272), *B. malayi* (MT674273- MT674275), *B. pahangi* (MT674276- MT674279), and *D. immitis* (MT674280- MT674288).

## 3 Results

### 3.1 Entomological findings from different ecotopes

A total of 1393 local mosquitoes, *Ar. subalbtus* (953), *Cx. vishnui* (133), *Cx. gelidus* (105), *Ae. albopictus* (80), *Ma. uniformis* (58), *Ae. aegypti* (23), *Ma. indiana* (15), *Ma. bonneae* (10), *Cq. crassipes* (6), *Cx. quinquefasciatus* (5), *Cx. sp.* (4), and *Cx. nigropunctatus* (1) were collected using the HLC method (Table 2). Based on the relative ratio (pi), *Ar. subalbatus* was the predominant species sessile to the elevated ecotopes, A (pi = 0.807), B (pi = 0.829), and C (pi = 0.543), but not the low-lying ecotope, D (pi = 0.103) when compared to other locally adapted mosquitoes such as *Cx. vishnui*, *Cx. gelidus*, *Ae. albopictus*, and *Ma. uniformis* (Table 2). The more abundant *Ar. subalbatus* mosquito population, which was locally adapted to the elevated ecotopes, A (29.67), B (31.83), and C (16.90), tended to exhibit an average MLR of 19.85, which was 4-fold to 20-fold greater than that of other mosquito species: *Culex* spp. (5.17), *Aedes* spp. (2.15), and *Mansonia* spp. (1.73) (Table 3).

**Table 2:** Species and numbers (p_i_)^a^ of human blood-seeking adult female mosquitoes^b^ in four ecotopes based HLC collection method, ordered by predominant species.

Species	Ecotope A (*N* = 441)		Ecotope B (*N* = 461)		Ecotope C (*N* = 374)		Ecotope D (*N* = 117)		All (*N* = 1393)	
	n	p_i_	n	p_i_	n	p_i_	n	p_i_	n	p_i_
*Ar. subalbatus*	356	0.807	382	0.829	203	0.543	12	0.103	953	0.684
*Cx. vishnui*	52	0.118	20	0.043	48	0.128	13	0.111	133	0.095
*Cx. gelidus*	11	0.025	13	0.028	69	0.184	12	0.102	105	0.075
*Ae. albopictus*	11	0.025	40	0.087	26	0.069	3	0.026	80	0.057
*Ma. uniformis*	1	0.002	2	0.004	3	0.008	52	0.444	58	0.042
*Ae. aegytpi*	5	0.011	3	0.006	10	0.027	5	0.043	23	0.016
*Ma. indiana*	2	0.005	0	0	10	0.027	3	0.026	15	0.011
*Ma. bonneae*	0	0	0	0	1	0.003	9	0.077	10	0.007
*Cq. crassipes*	3	0.007	0	0	3	0.008	0	0	6	0.004
*Cx. quinquefasciatus*	0	0	0	0	0	0	5	0.043	5	0.003
*Cx. sp.*	0	0	1	0.002	1	0.003	2	0.017	4	0.003
*Cx. nigropunctatus*	0	0	0	0	0	0	1	0.008	1	0.001

a The predominant species was derived based on the proportion of a mosquito species relative to the total number of all identified mosquito species (p_i_) present in each ecotope.

b The data were obtained from a repeated cross-sectional entomological survey between May 2014 and May 2015.

**Table 3:** The abundance of local mosquitoes in the four ecotopes, ordered by predominant local mosquitoes.^a^

Local mosquitoes	MLR^d^				
	Ecotope A	Ecotope B	Ecotope C	Ecotope D	Average
*Ar. subalbatus*	29.67	31.83	16.90	1.00	19.85
*Culex* spp. ^a^	5.25	2.83	9.83	2.75	5.17
*Aedes* spp.^b^	1.33	3.58	3.0	0.67	2.15
*Mansonia* spp.^c^	0.25	0.17	1.17	5.33	1.73
*Cq. crassipes*	0.25	0	0.25	0	0.12

a Including *Cx. vishnui*, *Cx. gelidus*, *Cx. quinquefasciatus*, *Cx. nigropunctatus*, and *Cx. sp.*

b Including *Ae. albopictus* and *Ae. aegypti*.

c Including *Ma. bonneae*, *Ma. uniformis*, and *Ma. indiana*.

d MLR expressed as the number of human blood-seeking mosquitoes per night per person.

All 440 HLC-collected adult female mosquitoes belonging to the genera *Culex* (*n* = 248), *Aedes* (*n* = 103), *Mansonia* (*n* = 83), and *Coquillettidia* (*n* = 6) (Table 2) were negative based on mosquito dissection. In contrast, of the 941 *Ar. subalbatus* mosquitoes obtained from the 9 *Ar. subalbatus* mosquito pools of the elevated ecotopes (A to C) alone, there were 24 infected mosquitoes (2.5% overall infection rate) of varying filarial parasite infections in ecotope C (2.96%), B (2.62%), and A (2.25%) (Table 4). However, *Ar. subalbatus* tended to exhibit an average infectious MLR of 0.5, with infectious MLRs of 0.83 (ecotope B), 0.67 (ecotope A), and 0.50 (ecotope C) (Table 4). All 9 *Ar. subalbatus* infectious pools had an average filarial larva number of 5 (110 larvae/24 infected mosquitoes). Further, *Ar. subalbatus* tended to harbor 56 infective L_3_, which was found to be three-fold greater than the amount of L_1_ (29) and L_2_ (25) harbored (Table S3).

**Table 4:** Infectious man landing rates (MLR) for *Armigeres subalbatus* in the four ecotopes.

Ecotope	Mosquito pool	Adult female no. (%)	Infectious adult female no. (% infection rate)	Infectious MLR^a^
A	AAP1	154	4	
	AAP2	79	3	
	AAP3	123	1	
	Subtotal	356 (37.36)	8 (2.25)	0.67
B	BAP1	155	3	
	BAP2	108	4	
	BAP3	119	3	
	Subtotal	382 (40.08)	10 (2.62)	0.83
C	CAP1	66	1	
	CAP2	45	2	
	CAP3	92	3	
	Subtotal	203 (21.30)	6 (2.96)	0.50
D	DAP1	8	0	
	DAP2	2	0	
	DAP3	2	0	
	Subtotal	12 (1.26)	0	0
All	Total	953	24 (2.52)^b^	0.50

a Infectious MLR expressed as the infectious number of human blood-seeking *Ar. subalbatus* per night per person for each ecotope.

b The overall infection rate with 95% CI (−0.17 to 4.08) for all ecotopes.

### 3.2 Molecular findings

**Fig. 4:**
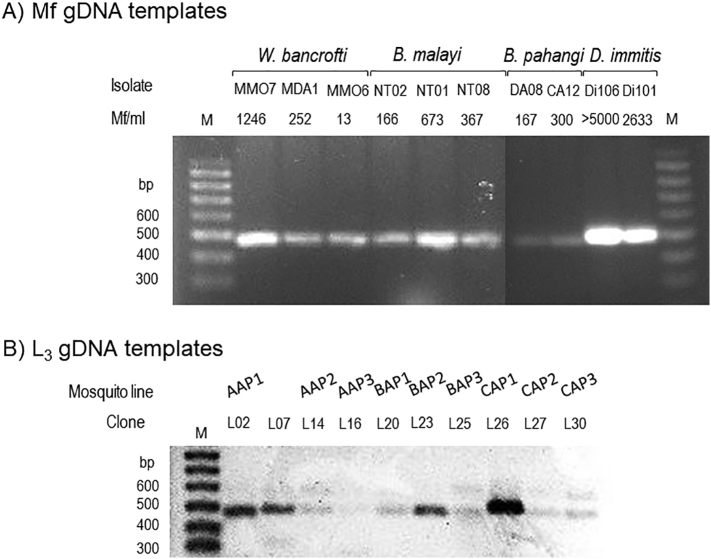
Touchup-nested PCR (TNPCR) amplification specific for orthologous *β-tubulin* genes of human and non-human filarial parasites using representative gDNA templates that were used to originally derive the Mf isolates (A) and L_3_ larva clones (B) as mentioned in the text. A) Amplification patterns of secondary TNPCR containing Mf gDNAs are displayed for specific amplicons with expected sizes (bp) authentically derived from the *β-tubulin* genes of *W. bancrofti* (493 bp), *B. malayi* (468 bp), *B. pahangi* (468 bp), and *D. immitis* (477 bp). B) Amplification patterns of secondary TNPCR containing L_3_ gDNAs are displayed for specific amplicons with expected sizes (bp): 468-bp *B. pahangi* amplicons (L02 and L30) and 477-bp *D. immitis* amplicons (L07, L14, L16, L20, L23, L25, L26, and L27).

Based on the orthologous *β-tubulin* gene-specific TUPCR amplification carried out using gDNA templates of 30 representative L_3_ larva clones, the *Ar. subalbatus* infectious pool, AAP1, corresponded to infections with both *B. pahangi* (468-bp amplicons of L02 clone) and *D. immitis* (477-bp amplicons of the L07 clone) (Fig. 4). The CAP3 infectious pool corresponded with the single infection of *B. pahangi*, showing 468-bp amplicons of the L28-L30 clones. The other infectious pools, AAP2, AAP3, BAP1, BAP2, BAP3, CAP1, and CAP2 might have carried the single infection of *D. immitis*, whose reactions putatively yielded 477-bp amplicons. None of the L_3_ gDNA was positive with the species-specific primers for *W. bancrofti* and *B. malayi*. Moreover, the representative L_3_ larva clones whose sequenced amplicons had 100% homology to the target filarial *β-tubulin* genes of *B. pahangi* were L02 and L30, while those of *D. immitis* were L07, L14, L20, L23, L25, L26, and L27.

## 4 Discussion

*Armigeres subalbatus*, the natural vector for *B. pahangi* and *D. immitis*, is commonly found in urban and rural areas in SEA [8,14,22,23] and South Asia [24,25]. In this study, *Ar. subalbatus* plantation ecotype was found to be the predominant species carrying zoonotic *B. pahangi* and *D. immitis*, and may have played an imperative role in circulating these parasites in the local landscape assessed in the rural area of Suratthani. Based on the results obtained herein, variation in the local landscape and environment influenced the infestation of *Ar. subalbatus*, enabling them to adapt well to the elevated ecotopes of rubber or oil palm plantations at 60–80 MASL. Rubber or oil palm plantations and not disturbed swamp, which was found in ecotope D, might be the ideal places for the breeding of *Ar. subalabatus* in both artificial and natural containers with organic matters. Its adaptability markedly contributed to its outnumbering of its counterpart species, such as *Mansonia*, *Aedes*, and *Culex,* and foraging blood meals in human dwellings with domestic cats or dogs. Further, there were high abundances of *Ar. subalbatus* in the elevated ecotopes as shown in Table 3 and Table S2.

In the local landscape examined herein, the zoonotic filarial infection rate was approximately 2.5% or up to 4% in the natural population of *Ar. subalbatus*. However, an accurate estimate of the *B. pahangi* infection in *Ar. subalbatus* was not established. When filarial orthologous beta-tubulin gene-specific TUPCR was carried out using representative L_3_ larva clones, any *Ar. subalbatus* infectious pool might have carried either *B. pahangi* or *D. immitis*, or both *B. pahangi* and *D. immitis*. This finding strongly suggests that *Ar. subalbatus* is susceptible to these zoonotic filarial parasites [8,[14], [15], [16]] with a wide range of infective L_3_ loads. *Ar. subalbatus* might have a disproportionate L_3_ load of *B. pahangi* relative to *D. immitis*, despite its infection rate being estimated to be less than 1% in the natural population. Such finding might be explained by the feeding behaviors of *Ar. subalbatus* that more likely carried out a vicious attack on dogs in the outdoor settings than cats when seeking animal blood meals during the peak hour of 18:30–19:30 h. Moreover, *Ar. subalbatus* might have a flight range longer than 100 m, even up to 1000 m. However, this might not relate *B. pahangi* infected cats to *B. pahangi* infections in *Ar. subalbatus* in the elevated ecotopes of A to C owing to its animal host range [23]. Similar to that observed in Malaysia, the local transmission of zoonotic *B. pahangi* might be due to the fitness of the *Ar. subalbatus* vectors, their ability to produce large numbers, and the source of infections in domestic cats [7,8,12]; a further study is required to verify the above findings.

Collectively, our findings are general considerations of the epidemiological catchment area comprised of designated sites scalable for vector surveillance and case investigation of *B. pahangi* infections in children. However, several factors, including the variation in the local landscape and environment, availability of *Ar. subalbatus* breeding sites proximal to the dwellings of domestic animals, the abundance of *Ar. subalbatus*, and the prevalence of *B. pahangi* infection in domestic animals, might be important.

## 5 Conclusion

Variations in the local landscape and environment, such as elevated instead of a low-lying ecotope of rubber plantations or oil palm plantations, could influence higher abundances of *Ar. subalbatus* plantation ecotypes and higher rates of zoonotic filarial parasite infections of *B. pahangi* and *D. immitis* in *Ar. subalbatus* than other local mosquitoes belonging to the genera *Mansonia*, *Aedes*, *Culex*, and *Coquillettidia*. Based on the molecular investigation of the L_3_ larva clones, which were representatives of the *Ar. subalbatus* infectious pools, *Ar. subalbatus* could either carry *B. pahangi* or *D. immitis*, or both species. Such findings imply the potential role of *Ar. subalbatus* in the natural transmission of not only zoonotic *B. pahangi* infection, but also *D. immitis* in plantation areas in Thailand, regardless of altitude.

## Ethics approval

The authors assert that all procedures contributing to this work complied with the ethical standards and were approved by the Institutional Review Board at the Faculty of Veterinary Medicine, Mahanakorn University of Technology (Approval No. ACUC-MUT-2014/001).

## Funding resources

The research was supported by The 10.13039/501100004396Thailand Research Fund; 10.13039/100012527Office of the Higher Education Commission; and 10.13039/501100001831Mahanakorn University of Technology, Bangkok, Thailand (grant number MRG5680087, 2013).

## Authors' contributions

Apiradee Intarapuk: Conceptualiation; Data curation; Formal analysis; Funding acquisition; Investigation; Methodology; Project administration; Resources; Validation; Writing - original draft.

Adisak Bhumiratana: Conceptualiation; Data curation; Formal analysis; Funding acquisition; Investigation; Methodology; Project administration; Resources; Validation; Visualization; Writing - original draft; Writing - review & editing.

## Declaration of Competing Interest

The authors declare that they have no known competing financial interests or personal relationships that could have appeared to influence the work reported in this paper.

## Appendix A Supplementary data

The Supplementary Table S1 Image 1

The Supplementary Table S2 Image 2

The Supplementary Table S3 Image 3

Amplicon sequences of L_3_ clones Image 4
