# Supplementary material for: Investigation of Armigeres subalbatus, a vector of zoonotic Brugia pahangi filariasis in plantation areas in Suratthani, Southern Thailand
Source: One Health. 2021 Apr 30;13:100261. doi: 10.1016/j.onehlt.2021.100261 (PMC8121957; doi:10.1016/j.onehlt.2021.100261)
Supplement: Supplementary file 1 — The Supplementary Table S1 [file mmc1.docx]

**Table S1** Current case reports of *B. pahangi* infection in children in Thailand^a^

| Case no | Year of diagnosis | Age (year) | Sex | Place | Local environmental conditions | | | Clinical features | Filarial infection status by thick blood film | Identification of *Brugia* species by PCR |
| --- | --- | --- | --- | --- | --- | --- | --- | --- | --- | --- |
|  |  |  |  |  | Landscape within a 100-m radius of patient house | Human blood-seeking mosquitoes within a 100-m radius of patient house | Proximity to *B. pahangi*-infected dogs or cats |  |  |  |
| 1 | 2012 | 2.0 | Male | Suratthani, Southern Thailand | Rubber and oil palm plantations with low altitude (40-45 m) | *Mansonia* (*Ma. uniformis* and *Ma. bonneae*), *Armigeres* *subalbatus*, *Anopheles* (*An. barbirostris* and *An. nigerrimus*);  *Culex* (*Cx. quinquefasciatus*, *Cx. gelidus*, and *Cx. vishui*) | 1-2 km | Fever without lymphatic pathology | Microfilaremia | *Brugia* sp. |
| 2 | 2013 | 1.5 | Male | Rayong, Eastern Thailand | Rubber plantations with low altitude (45-65 m) | *Mansonia* (*Ma. uniformis*); *Armigeres subalbatus*; *Anopheles* (*An. campestris*, *An. nitidus*, *An. barbirostris*, *An. umbrosus*)  *Culex* (*Cx. quinquefasciatus*, *Cx. gelidus*, *Cx. vishui*, *Cx. tritaenorhynchus*, and *Cx. fuscocephala*) | 1-2 km | Respiratory tract infections with neonatal cyanotic heart disease and inguinal lymphadenitis | Microfilaremia | *B. pahangi* |
| 3 | 2016 | 1.75 | Male | Suratthani, Southern Thailand | Rubber and oil palm plantations with low altitude (40-65 m) | N/A | N/A | Febrile convulsion without lymphatic pathology | Microfilaremia | *B. pahangi* |
| 4 | 2020 | 2.0 | Male | Rayong, Eastern Thailand | Rubber and oil palm plantations with low altitude (40-55 m) | *Culex* (*Cx. quinquefasciatus*); *Armigeres subalbatus*; *Mansonia indiana*; *Anopheles* (*An. campestris* and *An. barbirostris*); *Ae. albopictus* | N/A | Fever without lymphatic pathology | Microfilaremia | *B. pahangi* |

N/A - data not available.

^a^Sources of data or information for the cases were either obtained based on our own observations or were kindly provided by Dr. Wanapa Ritthison, Office of Disease Prevention and Control 11, Ministry of Public Health, Thailand.
