# Supplementary material for: Investigation of Armigeres subalbatus, a vector of zoonotic Brugia pahangi filariasis in plantation areas in Suratthani, Southern Thailand
Source: One Health. 2021 Apr 30;13:100261. doi: 10.1016/j.onehlt.2021.100261 (PMC8121957; doi:10.1016/j.onehlt.2021.100261)
Supplement: Supplementary file 2 — The Supplementary Table S2 [file mmc2.docx]

**Table S2** Local environmental conditions of the 100-m radius of the selected ecotopes

| **Ecotope** | **UTM grid zone and coordinate^a^ of mosquito sampling** | **Domestic animals surveyed^b^** | | **Availability of larval breeding habitats** |
| --- | --- | --- | --- | --- |
|  |  | **No. cats and dogs** | **No. of zoonotic filarial infections^c^** |  |
| A | 47P 509696 mE, 1056034 mN | 5 cats and 1 dog | 2 cats infected with *B. pahangi* | *Ar. subalbatus, Ae. albopictus*, and *Ae. agypti* |
| B | 47P 509644 mE, 1055593 mN | 1 cat and 1 dog | 1 cat infected with *B. pahangi* | *Ar. subalbatus*, *Ae. albopictus*, *Ae. agypti*, and *Culex* spp. |
| C | 47P 509661 mE, 1055313 mN | 1 cat and 2 dogs | 1 cat infected with *B. pahangi* | *Ar. subalbatus*, *Ae. albopictus*, *Ae. agypti*, and *Culex* spp. |
| D | 47P 508305 mE, 1054830 mN | 3 cats and 2 dogs | 1 cat and 1 dog infected with *B. pahangi*, 1 dog infected with *D. immitis* | *Ma. uniformis*, *Ma. indiana*, *Ae. albopictus*, *Ae. agypti*, and *Culex* spp. |

^a^ Numbers of UTM coordinates represent the measurement of the Easting (E) or Northing (N) position within a grid zone 47P, in meters (m).

^b^Surveyed numbers of domestic cats or dogs were obtained from a house used as the site of mosquito sampling.

^c^Either the number of *B. pahangi* or *D. immitis* infections was diagnosed using Giemsa-stained thick blood films.
