# Supplementary material for: Investigation of Armigeres subalbatus, a vector of zoonotic Brugia pahangi filariasis in plantation areas in Suratthani, Southern Thailand
Source: One Health. 2021 Apr 30;13:100261. doi: 10.1016/j.onehlt.2021.100261 (PMC8121957; doi:10.1016/j.onehlt.2021.100261)
Supplement: Supplementary file 3 — The Supplementary Table S3 [file mmc3.docx]

**Table S3** Filarial larva numbers obtained from the 9 *Armigeres subalbatus* infectious pools and representative L_3_ clones subjected to touchup-nested PCR

| **Pool no.** | **No. of infected mosquitoes**  **(n=24)** | **Filarial larva no.** | | | | **Average L_3_ no. per infected mosquito**  **(range)** | **No. of L_3_ used as gDNA templates**  **(n=30)** |
| --- | --- | --- | --- | --- | --- | --- | --- |
|  |  | **L_1_**  **(n=29)** | **L_2_**  **(n=25)** | **L_3_**  **(n=56)** | **Total**  **(n=110)** |  |  |
| AAP1 | 4 | 16 | 17 | 29 | 62 | 7.25 (2-12) | 12 |
| AAP2 | 3 | 0 | 0 | 6 | 6 | 2.00 (1-3) | 3 |
| AAP3 | 1 | 0 | 0 | 3 | 3 | 3.00 (0-3) | 3 |
| BAP1 | 3 | 1 | 0 | 8 | 9 | 2.67 (1-4) | 4 |
| BAP2 | 4 | 0 | 2 | 2 | 4 | 0.50 (0-1) | 1 |
| BAP3 | 3 | 0 | 1 | 4 | 5 | 1.33 (1-2) | 2 |
| CAP1 | 1 | 0 | 0 | 1 | 1 | 1.00 (0-1) | 1 |
| CAP2 | 2 | 8 | 4 | 1 | 13 | 0.50 (0-1) | 1 |
| CAP3 | 3 | 4 | 1 | 2 | 7 | 0.67 (0-3) | 3 |
| Average larvae no. per infected mosquito |  | 1.21 | 1.04 | 2.33^a^ | 4.58 |  |  |

^a^The average L_3_ load with 95% CI (-0.59 to 13.03) for the *Ar. subalbatus* infectious pools observed in the elevated ecotopes (A to C).
