# Supplementary material for: Investigation of Armigeres subalbatus, a vector of zoonotic Brugia pahangi filariasis in plantation areas in Suratthani, Southern Thailand
Source: One Health. 2021 Apr 30;13:100261. doi: 10.1016/j.onehlt.2021.100261 (PMC8121957; doi:10.1016/j.onehlt.2021.100261)
Supplement: Supplementary file 4 — Amplicon sequences of L3 clones [file mmc4.pdf]

SUMMARY OF GENBANK SEQUENCE SUBMISSION

Wuchereria bancrofti

Wuchereria bancrofti isolate MMO7 beta-tubulin isotype 1 (tub-1) gene, partial cds
GenBank: MT674270.1
https://www.ncbi.nlm.nih.gov/nuccore/MT674270

Wuchereria bancrofti isolate MDA1 beta-tubulin isotype 1 (tub-1) gene, partial cds
GenBank: MT674271.1
https://www.ncbi.nlm.nih.gov/nuccore/MT674271

Wuchereria bancrofti isolate MMO6 beta-tubulin isotype 1 (tub-1) gene, partial cds
GenBank: MT674272.1
https://www.ncbi.nlm.nih.gov/nuccore/MT674272

Brugia malayi

Brugia malayi isolate NT01 beta-tubulin isotype 1 (tub-1) gene, partial cds
GenBank: MT674273.1
https://www.ncbi.nlm.nih.gov/nuccore/MT674273

Brugia malayi isolate NT02 beta-tubulin isotype 1 (tub-1) gene, partial cds
GenBank: MT674274.1
https://www.ncbi.nlm.nih.gov/nuccore/MT674274

Brugia malayi isolate NT08 beta-tubulin isotype 1 (tub-1) gene, partial cds
GenBank: MT674275.1
https://www.ncbi.nlm.nih.gov/nuccore/MT674275

Brugia pahangi

Brugia pahangi isolate DA08 beta-tubulin isotype 1 (tub-1) gene, partial cds
GenBank: MT674276.1
https://www.ncbi.nlm.nih.gov/nuccore/MT674276

Brugia pahangi isolate CA12 beta-tubulin isotype 1 (tub-1) gene, partial cds
GenBank: MT674277.1
https://www.ncbi.nlm.nih.gov/nuccore/MT674277

Brugia pahangi isolate L02 beta-tubulin isotype 1 (tub-1) gene, partial cds
GenBank: MT674278.1
https://www.ncbi.nlm.nih.gov/nuccore/MT674278

Brugia pahangi isolate L30 beta-tubulin isotype 1 (tub-1) gene, partial cds
GenBank: MT674279.1
https://www.ncbi.nlm.nih.gov/nuccore/MT674279

Dirofilaria immitis

Dirofilaria immitis isolate Di106 beta-tubulin isotype 1 (tub-1) gene, partial cds
GenBank: MT674280.1
https://www.ncbi.nlm.nih.gov/nuccore/MT674280

Intarapuk A. Faculty of Veterinary Medicine, Mahanakorn University of Technology, 140 Chan Sampan rd., Nongchok, Bangkok 10530, Thailand

Dirofilaria immitis isolate Di101 beta-tubulin isotype 1 (tub-1) gene, partial cds
GenBank: MT674281.1
https://www.ncbi.nlm.nih.gov/nuccore/MT674281

Dirofilaria immitis isolate L07 beta-tubulin isotype 1 (tub-1) gene, partial cds
GenBank: MT674282.1
https://www.ncbi.nlm.nih.gov/nuccore/MT674282

Dirofilaria immitis isolate L14 beta-tubulin isotype 1 (tub-1) gene, partial cds
GenBank: MT674283.1
https://www.ncbi.nlm.nih.gov/nuccore/MT674283

Dirofilaria immitis isolate L20 beta-tubulin isotype 1 (tub-1) gene, partial cds
GenBank: MT674284.1
https://www.ncbi.nlm.nih.gov/nuccore/MT674284

Dirofilaria immitis isolate L23 beta-tubulin isotype 1 (tub-1) gene, partial cds
GenBank: MT674285.1
https://www.ncbi.nlm.nih.gov/nuccore/MT674285

Dirofilaria immitis isolate L25 beta-tubulin isotype 1 (tub-1) gene, partial cds
GenBank: MT674286.1
https://www.ncbi.nlm.nih.gov/nuccore/MT674286

Dirofilaria immitis isolate L26 beta-tubulin isotype 1 (tub-1) gene, partial cds
GenBank: MT674287.1
https://www.ncbi.nlm.nih.gov/nuccore/MT674287

Dirofilaria immitis isolate L27 beta-tubulin isotype 1 (tub-1) gene, partial cds
GenBank: MT674288.1
https://www.ncbi.nlm.nih.gov/nuccore/MT674288

SEQUENCE INFORMATION

```
Wuchereria bancrofti isolate MMO7 beta-tubulin isotype 1 (tub-1) gene, partial cds
GenBank: MT674270.1

FASTA Graphics
Go to:
LOCUS       MT674270                 450 bp    DNA     linear   INV 20-JAN-2021
DEFINITION  Wuchereria bancrofti isolate MMO7 beta-tubulin isotype 1 (tub-1)
            gene, partial cds.
ACCESSION   MT674270
VERSION     MT674270.1
KEYWORDS    .
SOURCE      Wuchereria bancrofti
  ORGANISM  Wuchereria bancrofti
            Eukaryota; Metazoa; Ecdysozoa; Nematoda; Chromadorea; Rhabditida;
            Spirurina; Spiruromorpha; Filarioidea; Onchocercidae; Wuchereria.
REFERENCE   1  (bases 1 to 450)
  AUTHORS   Intarapuk,A.
  TITLE     Direct Submission
  JOURNAL   Submitted (25-JUN-2020) Faculty of Veterinary Medicine, Mahanakorn
            University of Technology, 140 Chan Sampan rd., Nongchok, Bangkok
            10530, Thailand
COMMENT     ##Assembly-Data-START##
            Sequencing Technology :: Sanger dideoxy sequencing
            ##Assembly-Data-END##
FEATURES             Location/Qualifiers
     source          1..450
                     /organism="Wuchereria bancrofti"
                     /mol_type="genomic DNA"
                     /isolate="MMO7"
                     /db_xref="taxon:6293"
     gene            <1..>450
                     /gene="tub-1"
     mRNA            join(<1..114,305..>450)
                     /gene="tub-1"
                     /product="beta-tubulin isotype 1"
     CDS             join(<1..114,305..>450)
                     /gene="tub-1"
                     /codon_start=1
                     /product="beta-tubulin isotype 1"
                     /protein_id="QQP23390.1"
                     /translation="HSLGGGTGSGMGTLLISKIREEYPDRIMSSFSVVPSPKVSDVVL
                     EPYNATLSVHQLVENTDETFCIDNEALYDICFRTLKLANPTYG"
ORIGIN
        1 cattcacttg gtggcggtac cggttccggt atgggaacat tgctgatctc gaaaatccgt
       61 gaggagtatc cggatcgaat tatgagctct ttttcggttg tgccgtcgcc caaagtatgt
      121 atttgggttt ttgattatct tggttttaac atctgtttta tctactataa cgcataaaac
      181 atagctataa acatagctcc ataactttta agtggtgatt gtcgcacaaa gtggtctttt
      241 aactatcatt gcttcatttt catagttgaa gaaaaagtat ggtagacgta ttaatgattt
      301 ccaggtatca gatgttgtgt tggaacccta caatgcaaca ttatcagtcc accaactagt
      361 tgaaaacact gacgaaactt tctgcattga taacgaggct ttgtatgaca tctgcttccg
      421 aacgttgaag ttggcaaatc caacttacgg
//

Wuchereria bancrofti isolate MDA1 beta-tubulin isotype 1 (tub-1) gene, partial cds
GenBank: MT674271.1

FASTA Graphics
LOCUS       MT674271                 450 bp    DNA     linear   INV 20-JAN-2021
DEFINITION  Wuchereria bancrofti isolate MDA1 beta-tubulin isotype 1 (tub-1)
            gene, partial cds.
```

Intarapuk A. Faculty of Veterinary Medicine, Mahanakorn University of Technology, 140 Chan Sampan rd., Nongchok, Bangkok 10530, Thailand

```
ACCESSION   MT674271
VERSION     MT674271.1
KEYWORDS    .
SOURCE      Wuchereria bancrofti
  ORGANISM  Wuchereria bancrofti
            Eukaryota; Metazoa; Ecdysozoa; Nematoda; Chromadorea; Rhabditida;
            Spirurina; Spiruromorpha; Filarioidea; Onchocercidae; Wuchereria.
REFERENCE   1  (bases 1 to 450)
  AUTHORS   Intarapuk,A.
  TITLE     Direct Submission
  JOURNAL   Submitted (25-JUN-2020) Faculty of Veterinary Medicine, Mahanakorn
            University of Technology, 140 Chan Sampan rd., Nongchok, Bangkok
            10530, Thailand
COMMENT     ##Assembly-Data-START##
            Sequencing Technology :: Sanger dideoxy sequencing
            ##Assembly-Data-END##
FEATURES             Location/Qualifiers
     source          1..450
                     /organism="Wuchereria bancrofti"
                     /mol_type="genomic DNA"
                     /isolate="MDA1"
                     /db_xref="taxon:6293"
     gene            <1..>450
                     /gene="tub-1"
     mRNA            join(<1..114,305..>450)
                     /gene="tub-1"
                     /product="beta-tubulin isotype 1"
     CDS             join(<1..114,305..>450)
                     /gene="tub-1"
                     /codon_start=1
                     /product="beta-tubulin isotype 1"
                     /protein_id="QQP23391.1"
                     /translation="HSLGGGTGSGMGTLLISKIREEYPDRIMSSFSVVPSPKVSDVVL
                     EPYNATLSVHQLVENTDETFCIDNEALYDICFRTLKLANPTYG"
ORIGIN
        1 cattcacttg gtggcggtac cggttccggt atgggaacat tgctgatctc gaaaatccgt
       61 gaggagtatc cggatcgaat tatgagctct ttttcggttg tgccgtcgcc caaagtatgt
      121 atttgggttt ttgattatct tggttttaac atctgtttta tctactataa cgcataaaac
      181 atagctataa acatagctcc ataactttta agtggtgatt gtcgcacaaa gtggtctttt
      241 aactatcatt gcttcatttt catagttgaa gaaaaagtat ggtagacgta ttaatgattt
      301 ccaggtatca gatgttgtgt tggaacccta caatgcaaca ttatcagtcc accaactagt
      361 tgaaaacact gacgaaactt tctgcattga taacgaggct ttgtatgaca tctgcttccg
      421 aacgttgaag ttggcaaatc caacttacgg
//


Wuchereria bancrofti isolate MMO6 beta-tubulin isotype 1 (tub-1) gene, partial cds
GenBank: MT674272.1

FASTA Graphics
Go to:
LOCUS       MT674272                 450 bp    DNA     linear   INV 20-JAN-2021
DEFINITION  Wuchereria bancrofti isolate MMO6 beta-tubulin isotype 1 (tub-1)
            gene, partial cds.
ACCESSION   MT674272
VERSION     MT674272.1
KEYWORDS    .
SOURCE      Wuchereria bancrofti
  ORGANISM  Wuchereria bancrofti
            Eukaryota; Metazoa; Ecdysozoa; Nematoda; Chromadorea; Rhabditida;
            Spirurina; Spiruromorpha; Filarioidea; Onchocercidae; Wuchereria.
REFERENCE   1  (bases 1 to 450)
  AUTHORS   Intarapuk,A.
```

```
  TITLE     Direct Submission
  JOURNAL   Submitted (25-JUN-2020) Faculty of Veterinary Medicine, Mahanakorn
            University of Technology, 140 Chan Sampan rd., Nongchok, Bangkok
            10530, Thailand
COMMENT     ##Assembly-Data-START##
            Sequencing Technology :: Sanger dideoxy sequencing
            ##Assembly-Data-END##
FEATURES             Location/Qualifiers
     source          1..450
                     /organism="Wuchereria bancrofti"
                     /mol_type="genomic DNA"
                     /isolate="MMO6"
                     /db_xref="taxon:6293"
     gene            <1..>450
                     /gene="tub-1"
     mRNA            join(<1..114,305..>450)
                     /gene="tub-1"
                     /product="beta-tubulin isotype 1"
     CDS             join(<1..114,305..>450)
                     /gene="tub-1"
                     /codon_start=1
                     /product="beta-tubulin isotype 1"
                     /protein_id="QQP23392.1"
                     /translation="HSLGGGTGSGMGTLLISKIREEYPDRIMSSFSVVPSPKVSDVVL
                     EPYNATLSVHQLVENTDETFCIDNEALYDICFRTLKLANPTYG"
ORIGIN
        1 cattcacttg gtggcggtac cggttccggt atgggaacat tgctgatctc gaaaatccgt
       61 gaggagtatc cggatcgaat tatgagctct ttttcggttg tgccgtcgcc caaagtatgt
      121 atttgggttt ttgattatct tggttttaac atctgtttta tctactataa cgcataaaac
      181 atagctataa acatagctcc ataactttta agtggtgatt gtcgcacaaa gtggtctttt
      241 aactatcatt gcttcatttt catagttgaa gaaaaagtat ggtagacgta ttaatgattt
      301 ccaggtatca gatgttgtgt tggaacccta caatgcaaca ttatcagtcc accaactagt
      361 tgaaaacact gacgaaactt tctgcattga taacgaggct ttgtatgaca tctgcttccg
      421 aacgttgaag ttggcaaatc caacttacgg
//

Brugia malayi isolate NT01 beta-tubulin isotype 1 (tub-1) gene, partial cds
GenBank: MT674273.1

FASTA Graphics
LOCUS       MT674273                 425 bp    DNA     linear   INV 20-JAN-2021
DEFINITION  Brugia malayi isolate NT01 beta-tubulin isotype 1 (tub-1) gene,
            partial cds.
ACCESSION   MT674273
VERSION     MT674273.1
KEYWORDS    .
SOURCE      Brugia malayi
  ORGANISM  Brugia malayi
            Eukaryota; Metazoa; Ecdysozoa; Nematoda; Chromadorea; Rhabditida;
            Spirurina; Spiruromorpha; Filarioidea; Onchocercidae; Brugia.
REFERENCE   1  (bases 1 to 425)
  AUTHORS   Intarapuk,A.
  TITLE     Direct Submission
  JOURNAL   Submitted (26-JUN-2020) Faculty of Veterinary Medicine, Mahanakorn
            University of Technology, 140 Chan Sampan rd., Nongchok, Bangkok
            10530, Thailand
COMMENT     ##Assembly-Data-START##
            Sequencing Technology :: Sanger dideoxy sequencing
            ##Assembly-Data-END##
FEATURES             Location/Qualifiers
     source          1..425
                     /organism="Brugia malayi"
                     /mol_type="genomic DNA"
```

```
                     /isolate="NT01"
                     /db_xref="taxon:6279"
                     /PCR_primers="fwd_name: bt91, fwd_seq:
                     ggatccggatttcaactaacg, rev_name: bt123, rev_seq:
                     gaattccaaatggttgaggtca"
     gene            <1..>425
                     /gene="tub-1"
     mRNA            join(<1..114,280..>425)
                     /gene="tub-1"
                     /product="beta-tubulin isotype 1"
     CDS             join(<1..114,280..>425)
                     /gene="tub-1"
                     /codon_start=1
                     /product="beta-tubulin isotype 1"
                     /protein_id="QQP23393.1"
                     /translation="HSLGGGTGSGMGTLLISKIREEYPDRIMSSFSVVPSPKVSDVVL
                     EPYNATLSVHQLVENTDETFCIDNEALYDICFRTLKLANPTYG"
ORIGIN
        1 cattcacttg gtggtggtac cggttccggc atgggaacat tgctgatctc gaaaattcgt
       61 gaggagtatc cagatcgaat tatgagctct ttttcggttg tgccatcgcc caaagtatat
      121 atttgaattt ctgattctta tttttaacat ctgttatatc tattttaaac atagctccat
      181 aacttttaag tggcgattgt ctcacaaagt ggtttttcgg ctatcatatc attttcatag
      241 ttgaagaaaa agtatgctag acgtattaat gatttccagg tatcagatgt tgtgttggaa
      301 ccctacaatg caacattatc agtccaccaa ctagttgaaa acactgacga aactttctgc
      361 attgataacg aggctttgta tgacatctgc ttccgaacgt tgaagttggc aaatccaact
      421 tacgg
//

Brugia malayi isolate NT02 beta-tubulin isotype 1 (tub-1) gene, partial cds
GenBank: MT674274.1

FASTA Graphics
Go to:
LOCUS       MT674274                 425 bp    DNA     linear   INV 20-JAN-2021
DEFINITION  Brugia malayi isolate NT02 beta-tubulin isotype 1 (tub-1) gene,
            partial cds.
ACCESSION   MT674274
VERSION     MT674274.1
KEYWORDS    .
SOURCE      Brugia malayi
  ORGANISM  Brugia malayi
            Eukaryota; Metazoa; Ecdysozoa; Nematoda; Chromadorea; Rhabditida;
            Spirurina; Spiruromorpha; Filarioidea; Onchocercidae; Brugia.
REFERENCE   1  (bases 1 to 425)
  AUTHORS   Intarapuk,A.
  TITLE     Direct Submission
  JOURNAL   Submitted (26-JUN-2020) Faculty of Veterinary Medicine, Mahanakorn
            University of Technology, 140 Chan Sampan rd., Nongchok, Bangkok
            10530, Thailand
COMMENT     ##Assembly-Data-START##
            Sequencing Technology :: Sanger dideoxy sequencing
            ##Assembly-Data-END##
FEATURES             Location/Qualifiers
     source          1..425
                     /organism="Brugia malayi"
                     /mol_type="genomic DNA"
                     /isolate="NT02"
                     /db_xref="taxon:6279"
                     /PCR_primers="fwd_name: bt91, fwd_seq:
                     ggatccggatttcaactaacg, rev_name: bt123, rev_seq:
                     gaattccaaatggttgaggtca"
     gene            <1..>425
                     /gene="tub-1"
```

```
     mRNA            join(<1..114,280..>425)
                     /gene="tub-1"
                     /product="beta-tubulin isotype 1"
     CDS             join(<1..114,280..>425)
                     /gene="tub-1"
                     /codon_start=1
                     /product="beta-tubulin isotype 1"
                     /protein_id="QQP23394.1"
                     /translation="HSLGGGTGSGMGTLLISKIREEYPDRIMSSFSVVPSPKVSDVVL
                     EPYNATLSVHQLVENTDETFCIDNEALYDICFRTLKLANPTYG"
ORIGIN
        1 cattcacttg gtggtggtac cggttccggc atgggaacat tgctgatctc gaaaattcgt
       61 gaggagtatc cagatcgaat tatgagctct ttttcggttg tgccatcgcc caaagtatat
      121 atttgaattt ctgattctta tttttaacat ctgttatatc tattttaaac atagctccat
      181 aacttttaag tggcgattgt ctcacaaagt ggtttttcgg ctatcatatc attttcatag
      241 ttgaagaaaa agtatgctag acgtattaat gatttccagg tatcagatgt tgtgttggaa
      301 ccctacaatg caacattatc agtccaccaa ctagttgaaa acactgacga aactttctgc
      361 attgataacg aggctttgta tgacatctgc ttccgaacgt tgaagttggc aaatccaact
      421 tacgg
//

Brugia malayi isolate NT08 beta-tubulin isotype 1 (tub-1) gene, partial cds
GenBank: MT674275.1

FASTA Graphics
Go to:
LOCUS       MT674275                 425 bp    DNA     linear   INV 20-JAN-2021
DEFINITION  Brugia malayi isolate NT08 beta-tubulin isotype 1 (tub-1) gene,
            partial cds.
ACCESSION   MT674275
VERSION     MT674275.1
KEYWORDS    .
SOURCE      Brugia malayi
  ORGANISM  Brugia malayi
            Eukaryota; Metazoa; Ecdysozoa; Nematoda; Chromadorea; Rhabditida;
            Spirurina; Spiruromorpha; Filarioidea; Onchocercidae; Brugia.
REFERENCE   1  (bases 1 to 425)
  AUTHORS   Intarapuk,A.
  TITLE     Direct Submission
  JOURNAL   Submitted (26-JUN-2020) Faculty of Veterinary Medicine, Mahanakorn
            University of Technology, 140 Chan Sampan rd., Nongchok, Bangkok
            10530, Thailand
COMMENT     ##Assembly-Data-START##
            Sequencing Technology :: Sanger dideoxy sequencing
            ##Assembly-Data-END##
FEATURES             Location/Qualifiers
     source          1..425
                     /organism="Brugia malayi"
                     /mol_type="genomic DNA"
                     /isolate="NT08"
                     /db_xref="taxon:6279"
                     /PCR_primers="fwd_name: bt91, fwd_seq:
                     ggatccggatttcaactaacg, rev_name: bt123, rev_seq:
                     gaattccaaatggttgaggtca"
     gene            <1..>425
                     /gene="tub-1"
     mRNA            join(<1..114,280..>425)
                     /gene="tub-1"
                     /product="beta-tubulin isotype 1"
     CDS             join(<1..114,280..>425)
                     /gene="tub-1"
                     /codon_start=1
                     /product="beta-tubulin isotype 1"
```

```
                     /protein_id="QQP23395.1"
                     /translation="HSLGGGTGSGMGTLLISKIREEYPDRIMSSFSVVPSPKVSDVVL
                     EPYNATLSVHQLVENTDETFCIDNEALYDICFRTLKLANPTYG"
ORIGIN
        1 cattcacttg gtggtggtac cggttccggc atgggaacat tgctgatctc gaaaattcgt
       61 gaggagtatc cagatcgaat tatgagctct ttttcggttg tgccatcgcc caaagtatat
      121 atttgaattt ctgattctta tttttaacat ctgttatatc tattttaaac atagctccat
      181 aacttttaag tggcgattgt ctcacaaagt ggtttttcgg ctatcatatc attttcatag
      241 ttgaagaaaa agtatgctag acgtattaat gatttccagg tatcagatgt tgtgttggaa
      301 ccctacaatg caacattatc agtccaccaa ctagttgaaa acactgacga aactttctgc
      361 attgataacg aggctttgta tgacatctgc ttccgaacgt tgaagttggc aaatccaact
      421 tacgg
//
```

Brugia pahangi isolate DA08 beta-tubulin isotype 1 (tub-1) gene, partial cds
GenBank: MT674276.1

FASTA Graphics
Go to:

```
LOCUS       MT674276                 425 bp    DNA     linear   INV 20-JAN-2021
DEFINITION  Brugia pahangi isolate DA08 beta-tubulin isotype 1 (tub-1) gene,
            partial cds.
ACCESSION   MT674276
VERSION     MT674276.1
KEYWORDS    .
SOURCE      Brugia pahangi
  ORGANISM  Brugia pahangi
            Eukaryota; Metazoa; Ecdysozoa; Nematoda; Chromadorea; Rhabditida;
            Spirurina; Spiruromorpha; Filarioidea; Onchocercidae; Brugia.
REFERENCE   1  (bases 1 to 425)
  AUTHORS   Intarapuk,A.
  TITLE     Direct Submission
  JOURNAL   Submitted (26-JUN-2020) Faculty of Veterinary Medicine, Mahanakorn
            University of Technology, 140 Chan Sampan rd., Nongchok, Bangkok
            10530, Thailand
COMMENT     ##Assembly-Data-START##
            Sequencing Technology :: Sanger dideoxy sequencing
            ##Assembly-Data-END##
FEATURES             Location/Qualifiers
     source          1..425
                     /organism="Brugia pahangi"
                     /mol_type="genomic DNA"
                     /isolate="DA08"
                     /db_xref="taxon:6280"
                     /PCR_primers="fwd_name: bt91, fwd_seq:
                     ggatccggatttcaactaacg, rev_name: bt123, rev_seq:
                     gaattccaaatggttgaggtca"
     gene            <1..>425
                     /gene="tub-1"
     mRNA            join(<1..114,280..>425)
                     /gene="tub-1"
                     /product="beta-tubulin isotype 1"
     CDS             join(<1..114,280..>425)
                     /gene="tub-1"
                     /codon_start=1
                     /product="beta-tubulin isotype 1"
                     /protein_id="QQP23396.1"
                     /translation="HSLGGGTGSGMGTLLISKIREEYPDRIMSSFSVVPSPKVSDVVL
                     EPYNATLSVHQLVENTDETFCIDNEALYDICFRTLKLANPTYG"
ORIGIN
        1 cattcacttg gtggtggtac cggttccggc atgggaacat tgctgatctc gaaaattcgt
       61 gaggagtatc cggatcgaat tatgagctct ttttcggttg tgccatcgcc caaagtatgt
      121 atttgaattt ctgattctta tttttaacat ctgttatatc tattttaaac atagctccat
```

```
      181 aacttttaag tggcggttgt ctcacaaagt ggtttttcgg ctatcatttc attttcatag
      241 ttgaagaaaa agtatgctag acgtattaat gatttccagg tatcagatgt tgtgttggaa
      301 ccctacaatg caacattatc agtccaccaa ctagttgaaa acactgacga aactttctgc
      361 attgataacg aggctttgta tgacatctgc ttccgaacgt tgaagttggc aaatccaact
      421 tacgg
//
```

```
Brugia pahangi isolate CA12 beta-tubulin isotype 1 (tub-1) gene, partial cds
GenBank: MT674277.1

FASTA Graphics
Go to:
LOCUS       MT674277                 425 bp    DNA     linear   INV 20-JAN-2021
DEFINITION  Brugia pahangi isolate CA12 beta-tubulin isotype 1 (tub-1) gene,
            partial cds.
ACCESSION   MT674277
VERSION     MT674277.1
KEYWORDS    .
SOURCE      Brugia pahangi
  ORGANISM  Brugia pahangi
            Eukaryota; Metazoa; Ecdysozoa; Nematoda; Chromadorea; Rhabditida;
            Spirurina; Spiruromorpha; Filarioidea; Onchocercidae; Brugia.
REFERENCE   1  (bases 1 to 425)
  AUTHORS   Intarapuk,A.
  TITLE     Direct Submission
  JOURNAL   Submitted (26-JUN-2020) Faculty of Veterinary Medicine, Mahanakorn
            University of Technology, 140 Chan Sampan rd., Nongchok, Bangkok
            10530, Thailand
COMMENT     ##Assembly-Data-START##
            Sequencing Technology :: Sanger dideoxy sequencing
            ##Assembly-Data-END##
FEATURES             Location/Qualifiers
     source          1..425
                     /organism="Brugia pahangi"
                     /mol_type="genomic DNA"
                     /isolate="CA12"
                     /db_xref="taxon:6280"
                     /PCR_primers="fwd_name: bt91, fwd_seq:
                     ggatccggatttcaactaacg, rev_name: bt123, rev_seq:
                     gaattccaaatggttgaggtca"
     gene            <1..>425
                     /gene="tub-1"
     mRNA            join(<1..114,280..>425)
                     /gene="tub-1"
                     /product="beta-tubulin isotype 1"
     CDS             join(<1..114,280..>425)
                     /gene="tub-1"
                     /codon_start=1
                     /product="beta-tubulin isotype 1"
                     /protein_id="QQP23397.1"
                     /translation="HSLGGGTGSGMGTLLISKIREEYPDRIMSSFSVVPSPKVSDVVL
                     EPYNATLSVHQLVENTDETFCIDNEALYDICFRTLKLANPTYG"
ORIGIN
        1 cattcacttg gtggtggtac cggttccggc atgggaacat tgctgatctc gaaaattcgt
       61 gaggagtatc cggatcgaat tatgagctct ttttcggttg tgccatcgcc caaagtatgt
      121 atttgaattt ctgattctta tttttaacat ctgttatatc tattttaaac atagctccat
      181 aacttttaag tggcggttgt ctcacaaagt ggtttttcgg ctatcatttc attttcatag
      241 ttgaagaaaa agtatgctag acgtattaat gatttccagg tatcagatgt tgtgttggaa
      301 ccctacaatg caacattatc agtccaccaa ctagttgaaa acactgacga aactttctgc
      361 attgataacg aggctttgta tgacatctgc ttccgaacgt tgaagttggc aaatccaact
      421 tacgg
//
```

```
Brugia pahangi isolate L02 beta-tubulin isotype 1 (tub-1) gene, partial cds
GenBank: MT674278.1

FASTA Graphics
Go to:
LOCUS       MT674278                 425 bp    DNA     linear   INV 20-JAN-2021
DEFINITION  Brugia pahangi isolate L02 beta-tubulin isotype 1 (tub-1) gene,
            partial cds.
ACCESSION   MT674278
VERSION     MT674278.1
KEYWORDS    .
SOURCE      Brugia pahangi
  ORGANISM  Brugia pahangi
            Eukaryota; Metazoa; Ecdysozoa; Nematoda; Chromadorea; Rhabditida;
            Spirurina; Spiruromorpha; Filarioidea; Onchocercidae; Brugia.
REFERENCE   1  (bases 1 to 425)
  AUTHORS   Intarapuk,A.
  TITLE     Direct Submission
  JOURNAL   Submitted (26-JUN-2020) Faculty of Veterinary Medicine, Mahanakorn
            University of Technology, 140 Chan Sampan rd., Nongchok, Bangkok
            10530, Thailand
COMMENT     ##Assembly-Data-START##
            Sequencing Technology :: Sanger dideoxy sequencing
            ##Assembly-Data-END##
FEATURES             Location/Qualifiers
     source          1..425
                     /organism="Brugia pahangi"
                     /mol_type="genomic DNA"
                     /isolate="L02"
                     /db_xref="taxon:6280"
                     /PCR_primers="fwd_name: bt91, fwd_seq:
                     ggatccggatttcaactaacg, rev_name: bt123, rev_seq:
                     gaattccaaatggttgaggtca"
     gene            <1..>425
                     /gene="tub-1"
     mRNA            join(<1..114,280..>425)
                     /gene="tub-1"
                     /product="beta-tubulin isotype 1"
     CDS             join(<1..114,280..>425)
                     /gene="tub-1"
                     /codon_start=1
                     /product="beta-tubulin isotype 1"
                     /protein_id="QQP23398.1"
                     /translation="HSLGGGTGSGMGTLLISKIREEYPDRIMSSFSVVPSPKVSDVVL
                     EPYNATLSVHQLVENTDETFCIDNEALYDICFRTLKLANPTYG"
ORIGIN
        1 cattcacttg gtggtggtac cggttccggc atgggaacat tgctgatctc gaaaattcgt
       61 gaggagtatc cggatcgaat tatgagctct ttttcggttg tgccatcgcc caaagtatgt
      121 atttgaattt ctgattctta tttttaacat ctgttatatc tattttaaac atagctccat
      181 aacttttaag tggcggttgt ctcacaaagt ggtttttcgg ctatcatttc attttcatag
      241 ttgaagaaaa agtatgctag acgtattaat gatttccagg tatcagatgt tgtgttggaa
      301 ccctacaatg caacattatc agtccaccaa ctagttgaaa acactgacga aactttctgc
      361 attgataacg aggctttgta tgacatctgc ttccgaacgt tgaagttggc aaatccaact
      421 tacgg
//

Brugia pahangi isolate L30 beta-tubulin isotype 1 (tub-1) gene, partial cds
GenBank: MT674279.1

FASTA Graphics
Go to:
LOCUS       MT674279                 425 bp    DNA     linear   INV 20-JAN-2021
DEFINITION  Brugia pahangi isolate L30 beta-tubulin isotype 1 (tub-1) gene,
```

```
             partial cds.
ACCESSION   MT674279
VERSION     MT674279.1
KEYWORDS    .
SOURCE      Brugia pahangi
  ORGANISM  Brugia pahangi
            Eukaryota; Metazoa; Ecdysozoa; Nematoda; Chromadorea; Rhabditida;
            Spirurina; Spiruromorpha; Filarioidea; Onchocercidae; Brugia.
REFERENCE   1  (bases 1 to 425)
  AUTHORS   Intarapuk,A.
  TITLE     Direct Submission
  JOURNAL   Submitted (26-JUN-2020) Faculty of Veterinary Medicine, Mahanakorn
            University of Technology, 140 Chan Sampan rd., Nongchok, Bangkok
            10530, Thailand
COMMENT     ##Assembly-Data-START##
            Sequencing Technology :: Sanger dideoxy sequencing
            ##Assembly-Data-END##
FEATURES             Location/Qualifiers
     source          1..425
                     /organism="Brugia pahangi"
                     /mol_type="genomic DNA"
                     /isolate="L30"
                     /db_xref="taxon:6280"
                     /PCR_primers="fwd_name: bt91, fwd_seq:
                     ggatccggatttcaactaacg, rev_name: bt123, rev_seq:
                     gaattccaaatggttgaggtca"
     gene            <1..>425
                     /gene="tub-1"
     mRNA            join(<1..114,280..>425)
                     /gene="tub-1"
                     /product="beta-tubulin isotype 1"
     CDS             join(<1..114,280..>425)
                     /gene="tub-1"
                     /codon_start=1
                     /product="beta-tubulin isotype 1"
                     /protein_id="QQP23399.1"
                     /translation="HSLGGGTGSGMGTLLISKIREEYPDRIMSSFSVVPSPKVSDVVL
                     EPYNATLSVHQLVENTDETFCIDNEALYDICFRTLKLANPTYG"
ORIGIN
        1 cattcacttg gtggtggtac cggttccggc atgggaacat tgctgatctc gaaaattcgt
       61 gaggagtatc cggatcgaat tatgagctct ttttcggttg tgccatcgcc caaagtatgt
      121 atttgaattt ctgattctta tttttaacat ctgttatatc tattttaaac atagctccat
      181 aacttttaag tggcggttgt ctcacaaagt ggtttttcgg ctatcatttc attttcatag
      241 ttgaagaaaa agtatgctag acgtattaat gatttccagg tatcagatgt tgtgttggaa
      301 ccctacaatg caacattatc agtccaccaa ctagttgaaa acactgacga aactttctgc
      361 attgataacg aggctttgta tgacatctgc ttccgaacgt tgaagttggc aaatccaact
      421 tacgg
//
```

Dirofilaria immitis isolate Di106 beta-tubulin isotype 1 (tub-1) gene, partial cds
GenBank: MT674280.1

FASTA Graphics
Go to:

```
LOCUS       MT674280                 434 bp    DNA     linear   INV 20-JAN-2021
DEFINITION  Dirofilaria immitis isolate Di106 beta-tubulin isotype 1 (tub-1)
            gene, partial cds.
ACCESSION   MT674280
VERSION     MT674280.1
KEYWORDS    .
SOURCE      Dirofilaria immitis (dog heartworm nematode)
  ORGANISM  Dirofilaria immitis
            Eukaryota; Metazoa; Ecdysozoa; Nematoda; Chromadorea; Rhabditida;
```

```
             Spirurina; Spiruromorpha; Filarioidea; Onchocercidae; Dirofilaria.
REFERENCE   1  (bases 1 to 434)
  AUTHORS   Intarapuk,A.
  TITLE     Direct Submission
  JOURNAL   Submitted (26-JUN-2020) Faculty of Veterinary Medicine, Mahanakorn
            University of Technology, 140 Chan Sampan rd., Nongchok, Bangkok
            10530, Thailand
COMMENT     ##Assembly-Data-START##
            Sequencing Technology :: Sanger dideoxy sequencing
            ##Assembly-Data-END##
FEATURES             Location/Qualifiers
     source          1..434
                     /organism="Dirofilaria immitis"
                     /mol_type="genomic DNA"
                     /isolate="Di106"
                     /db_xref="taxon:6287"
                     /PCR_primers="fwd_name: bt93, fwd_seq:
                     ggatccggattccaactgact, rev_name: bt26, rev_seq:
                     gaattccaagtgattgagatcg"
     gene            <1..>434
                     /gene="tub-1"
     mRNA            join(<1..114,289..>434)
                     /gene="tub-1"
                     /product="beta-tubulin isotype 1"
     CDS             join(<1..114,289..>434)
                     /gene="tub-1"
                     /codon_start=1
                     /product="beta-tubulin isotype 1"
                     /protein_id="QQP23400.1"
                     /translation="HSLGGGTGSGMGTLLISKIREEYPDRIMSSFSVVPSPKVSDVVL
                     EPYNATLSVHQLVENTDETFCIDNEALYDICFRTLKLTNPTYG"
ORIGIN
        1 cattcacttg gaggtggtac aggttctggt atgggaacat tgcttatctc gaagatccgt
       61 gaggaatatc cagatcggat tatgagctct ttttcggttg tgccatcacc taaagtatgt
      121 atatttgtgt cttaactagt ttgatttaat tttcttggtt catacctttt cgttatttag
      181 aagccatcat tttatttgct tcaaattcat gaatgaagtg aacactatct gacgagatga
      241 ttttaatctg tccctatttc cttttgaaat gcaaggttgg tttttcaggt atcagatgtt
      301 gtgttggaac cttacaatgc aacgttatca gtgcatcaat tagttgaaaa cactgatgaa
      361 actttctgca ttgataatga agctttatat gatatctgct tccgaacatt gaaattgacg
      421 aatccaactt acgg
//

Dirofilaria immitis isolate Di101 beta-tubulin isotype 1 (tub-1) gene, partial cds
GenBank: MT674281.1

FASTA Graphics
Go to:
LOCUS       MT674281                 434 bp    DNA     linear   INV 20-JAN-2021
DEFINITION  Dirofilaria immitis isolate Di101 beta-tubulin isotype 1 (tub-1)
            gene, partial cds.
ACCESSION   MT674281
VERSION     MT674281.1
KEYWORDS    .
SOURCE      Dirofilaria immitis (dog heartworm nematode)
  ORGANISM  Dirofilaria immitis
            Eukaryota; Metazoa; Ecdysozoa; Nematoda; Chromadorea; Rhabditida;
            Spirurina; Spiruromorpha; Filarioidea; Onchocercidae; Dirofilaria.
REFERENCE   1  (bases 1 to 434)
  AUTHORS   Intarapuk,A.
  TITLE     Direct Submission
  JOURNAL   Submitted (26-JUN-2020) Faculty of Veterinary Medicine, Mahanakorn
            University of Technology, 140 Chan Sampan rd., Nongchok, Bangkok
            10530, Thailand
```

```
COMMENT     ##Assembly-Data-START##
            Sequencing Technology :: Sanger dideoxy sequencing
            ##Assembly-Data-END##
FEATURES             Location/Qualifiers
     source          1..434
                     /organism="Dirofilaria immitis"
                     /mol_type="genomic DNA"
                     /isolate="Di101"
                     /db_xref="taxon:6287"
                     /PCR_primers="fwd_name: bt93, fwd_seq:
                     ggatccggattccaactgact, rev_name: bt26, rev_seq:
                     gaattccaagtgattgagatcg"
     gene            <1..>434
                     /gene="tub-1"
     mRNA            join(<1..114,289..>434)
                     /gene="tub-1"
                     /product="beta-tubulin isotype 1"
     CDS             join(<1..114,289..>434)
                     /gene="tub-1"
                     /codon_start=1
                     /product="beta-tubulin isotype 1"
                     /protein_id="QQP23401.1"
                     /translation="HSLGGGTGSGMGTLLISKIREEYPDRIMSSFSVVPSPKVSDVVL
                     EPYNATLSVHQLVENTDETFCIDNEALYDICFRTLKLTNPTYG"
ORIGIN
        1 cattcacttg gaggtggtac aggttctggt atgggaacat tgcttatctc gaagatccgt
       61 gaggaatatc cagatcggat tatgagctct ttttcggttg tgccatcacc taaagtatgt
      121 atatttgtgt cttaactagt ttgatttaat tttcttggtt catacctttt cgttatttag
      181 aagccatcat tttatttgct tcaaattcat gaatgaagtg aacactatct gacgagatga
      241 ttttaatctg tccctatttc cttttgaaat gcaaggttgg tttttcaggt atcagatgtt
      301 gtgttggaac cttacaatgc aacgttatca gtgcatcaat tagttgaaaa cactgatgaa
      361 actttctgca ttgataatga agctttatat gatatctgct tccgaacatt gaaattgacg
      421 aatccaactt acgg
//

Dirofilaria immitis isolate L07 beta-tubulin isotype 1 (tub-1) gene, partial cds
GenBank: MT674282.1

FASTA Graphics
Go to:
LOCUS       MT674282                 434 bp    DNA     linear   INV 20-JAN-2021
DEFINITION  Dirofilaria immitis isolate L07 beta-tubulin isotype 1 (tub-1)
            gene, partial cds.
ACCESSION   MT674282
VERSION     MT674282.1
KEYWORDS    .
SOURCE      Dirofilaria immitis (dog heartworm nematode)
  ORGANISM  Dirofilaria immitis
            Eukaryota; Metazoa; Ecdysozoa; Nematoda; Chromadorea; Rhabditida;
            Spirurina; Spiruromorpha; Filarioidea; Onchocercidae; Dirofilaria.
REFERENCE   1  (bases 1 to 434)
  AUTHORS   Intarapuk,A.
  TITLE     Direct Submission
  JOURNAL   Submitted (26-JUN-2020) Faculty of Veterinary Medicine, Mahanakorn
            University of Technology, 140 Chan Sampan rd., Nongchok, Bangkok
            10530, Thailand
COMMENT     ##Assembly-Data-START##
            Sequencing Technology :: Sanger dideoxy sequencing
            ##Assembly-Data-END##
FEATURES             Location/Qualifiers
     source          1..434
                     /organism="Dirofilaria immitis"
                     /mol_type="genomic DNA"
```

```
                     /isolate="L07"
                     /db_xref="taxon:6287"
                     /PCR_primers="fwd_name: bt93, fwd_seq:
                     ggatccggattccaactgact, rev_name: bt26, rev_seq:
                     gaattccaagtgattgagatcg"
     gene            <1..>434
                     /gene="tub-1"
     mRNA            join(<1..114,289..>434)
                     /gene="tub-1"
                     /product="beta-tubulin isotype 1"
     CDS             join(<1..114,289..>434)
                     /gene="tub-1"
                     /codon_start=1
                     /product="beta-tubulin isotype 1"
                     /protein_id="QQP23402.1"
                     /translation="HSLGGGTGSGMGTLLISKIREEYPDRIMSSFSVVPSPKVSDVVL
                     EPYNATLSVHQLVENTDETFCIDNEALYDICFRTLKLTNPTYG"
ORIGIN
        1 cattcacttg gaggtggtac aggttctggt atgggaacat tgcttatctc gaagatccgt
       61 gaggaatatc cagatcggat tatgagctct ttttcggttg tgccatcacc taaagtatgt
      121 atatttgtgt cttaactagt ttgatttaat tttcttggtt catacctttt cgttatttag
      181 aagccatcat tttatttgct tcaaattcat gaatgaagtg aacactatct gacgagatga
      241 ttttaatctg tccctatttc cttttgaaat gcaaggttgg tttttcaggt atcagatgtt
      301 gtgttggaac cttacaatgc aacgttatca gtgcatcaat tagttgaaaa cactgatgaa
      361 actttctgca ttgataatga agctttatat gatatctgct tccgaacatt gaaattgacg
      421 aatccaactt acgg
//

Dirofilaria immitis isolate L14 beta-tubulin isotype 1 (tub-1) gene, partial cds
GenBank: MT674283.1

FASTA Graphics
Go to:
LOCUS       MT674283                 434 bp    DNA     linear   INV 20-JAN-2021
DEFINITION  Dirofilaria immitis isolate L14 beta-tubulin isotype 1 (tub-1)
            gene, partial cds.
ACCESSION   MT674283
VERSION     MT674283.1
KEYWORDS    .
SOURCE      Dirofilaria immitis (dog heartworm nematode)
  ORGANISM  Dirofilaria immitis
            Eukaryota; Metazoa; Ecdysozoa; Nematoda; Chromadorea; Rhabditida;
            Spirurina; Spiruromorpha; Filarioidea; Onchocercidae; Dirofilaria.
REFERENCE   1  (bases 1 to 434)
  AUTHORS   Intarapuk,A.
  TITLE     Direct Submission
  JOURNAL   Submitted (26-JUN-2020) Faculty of Veterinary Medicine, Mahanakorn
            University of Technology, 140 Chan Sampan rd., Nongchok, Bangkok
            10530, Thailand
COMMENT     ##Assembly-Data-START##
            Sequencing Technology :: Sanger dideoxy sequencing
            ##Assembly-Data-END##
FEATURES             Location/Qualifiers
     source          1..434
                     /organism="Dirofilaria immitis"
                     /mol_type="genomic DNA"
                     /isolate="L14"
                     /db_xref="taxon:6287"
                     /PCR_primers="fwd_name: bt93, fwd_seq:
                     ggatccggattccaactgact, rev_name: bt26, rev_seq:
                     gaattccaagtgattgagatcg"
     gene            <1..>434
                     /gene="tub-1"
```

```
     mRNA            join(<1..114,289..>434)
                     /gene="tub-1"
                     /product="beta-tubulin isotype 1"
     CDS             join(<1..114,289..>434)
                     /gene="tub-1"
                     /codon_start=1
                     /product="beta-tubulin isotype 1"
                     /protein_id="QQP23403.1"
                     /translation="HSLGGGTGSGMGTLLISKIREEYPDRIMSSFSVVPSPKVSDVVL
                     EPYNATLSVHQLVENTDETFCIDNEALYDICFRTLKLTNPTYG"
ORIGIN
        1 cattcacttg gaggtggtac aggttctggt atgggaacat tgcttatctc gaagatccgt
       61 gaggaatatc cagatcggat tatgagctct ttttcggttg tgccatcacc taaagtatgt
      121 atatttgtgt cttaactagt ttgatttaat tttcttggtt catacctttt cgttatttag
      181 aagccatcat tttatttgct tcaaattcat gaatgaagtg aacactatct gacgagatga
      241 ttttaatctg tccctatttc cttttgaaat gcaaggttgg tttttcaggt atcagatgtt
      301 gtgttggaac cttacaatgc aacgttatca gtgcatcaat tagttgaaaa cactgatgaa
      361 actttctgca ttgataatga agctttatat gatatctgct tccgaacatt gaaattgacg
      421 aatccaactt acgg
//

Dirofilaria immitis isolate L20 beta-tubulin isotype 1 (tub-1) gene, partial cds
GenBank: MT674284.1

FASTA Graphics
Go to:
LOCUS       MT674284                 434 bp    DNA     linear   INV 20-JAN-2021
DEFINITION  Dirofilaria immitis isolate L20 beta-tubulin isotype 1 (tub-1)
            gene, partial cds.
ACCESSION   MT674284
VERSION     MT674284.1
KEYWORDS    .
SOURCE      Dirofilaria immitis (dog heartworm nematode)
  ORGANISM  Dirofilaria immitis
            Eukaryota; Metazoa; Ecdysozoa; Nematoda; Chromadorea; Rhabditida;
            Spirurina; Spiruromorpha; Filarioidea; Onchocercidae; Dirofilaria.
REFERENCE   1  (bases 1 to 434)
  AUTHORS   Intarapuk,A.
  TITLE     Direct Submission
  JOURNAL   Submitted (26-JUN-2020) Faculty of Veterinary Medicine, Mahanakorn
            University of Technology, 140 Chan Sampan rd., Nongchok, Bangkok
            10530, Thailand
COMMENT     ##Assembly-Data-START##
            Sequencing Technology :: Sanger dideoxy sequencing
            ##Assembly-Data-END##
FEATURES             Location/Qualifiers
     source          1..434
                     /organism="Dirofilaria immitis"
                     /mol_type="genomic DNA"
                     /isolate="L20"
                     /db_xref="taxon:6287"
                     /PCR_primers="fwd_name: bt93, fwd_seq:
                     ggatccggattccaactgact, rev_name: bt26, rev_seq:
                     gaattccaagtgattgagatcg"
     gene            <1..>434
                     /gene="tub-1"
     mRNA            join(<1..114,289..>434)
                     /gene="tub-1"
                     /product="beta-tubulin isotype 1"
     CDS             join(<1..114,289..>434)
                     /gene="tub-1"
                     /codon_start=1
                     /product="beta-tubulin isotype 1"
```

```
                     /protein_id="QQP23404.1"
                     /translation="HSLGGGTGSGMGTLLISKIREEYPDRIMSSFSVVPSPKVSDVVL
                     EPYNATLSVHQLVENTDETFCIDNEALYDICFRTLKLTNPTYG"
ORIGIN
        1 cattcacttg gaggtggtac aggttctggt atgggaacat tgcttatctc gaagatccgt
       61 gaggaatatc cagatcggat tatgagctct ttttcggttg tgccatcacc taaagtatgt
      121 atatttgtgt cttaactagt ttgatttaat tttcttggtt catacctttt cgttatttag
      181 aagccatcat tttatttgct tcaaattcat gaatgaagtg aacactatct gacgagatga
      241 ttttaatctg tccctatttc cttttgaaat gcaaggttgg tttttcaggt atcagatgtt
      301 gtgttggaac cttacaatgc aacgttatca gtgcatcaat tagttgaaaa cactgatgaa
      361 actttctgca ttgataatga agctttatat gatatctgct tccgaacatt gaaattgacg
      421 aatccaactt acgg
//

Dirofilaria immitis isolate L23 beta-tubulin isotype 1 (tub-1) gene, partial cds
GenBank: MT674285.1

FASTA Graphics
Go to:
LOCUS       MT674285                 434 bp    DNA     linear   INV 20-JAN-2021
DEFINITION  Dirofilaria immitis isolate L23 beta-tubulin isotype 1 (tub-1)
            gene, partial cds.
ACCESSION   MT674285
VERSION     MT674285.1
KEYWORDS    .
SOURCE      Dirofilaria immitis (dog heartworm nematode)
  ORGANISM  Dirofilaria immitis
            Eukaryota; Metazoa; Ecdysozoa; Nematoda; Chromadorea; Rhabditida;
            Spirurina; Spiruromorpha; Filarioidea; Onchocercidae; Dirofilaria.
REFERENCE   1  (bases 1 to 434)
  AUTHORS   Intarapuk,A.
  TITLE     Direct Submission
  JOURNAL   Submitted (26-JUN-2020) Faculty of Veterinary Medicine, Mahanakorn
            University of Technology, 140 Chan Sampan rd., Nongchok, Bangkok
            10530, Thailand
COMMENT     ##Assembly-Data-START##
            Sequencing Technology :: Sanger dideoxy sequencing
            ##Assembly-Data-END##
FEATURES             Location/Qualifiers
     source          1..434
                     /organism="Dirofilaria immitis"
                     /mol_type="genomic DNA"
                     /isolate="L23"
                     /db_xref="taxon:6287"
                     /PCR_primers="fwd_name: bt93, fwd_seq:
                     ggatccggattccaactgact, rev_name: bt26, rev_seq:
                     gaattccaagtgattgagatcg"
     gene            <1..>434
                     /gene="tub-1"
     mRNA            join(<1..114,289..>434)
                     /gene="tub-1"
                     /product="beta-tubulin isotype 1"
     CDS             join(<1..114,289..>434)
                     /gene="tub-1"
                     /codon_start=1
                     /product="beta-tubulin isotype 1"
                     /protein_id="QQP23405.1"
                     /translation="HSLGGGTGSGMGTLLISKIREEYPDRIMSSFSVVPSPKVSDVVL
                     EPYNATLSVHQLVENTDETFCIDNEALYDICFRTLKLTNPTYG"
ORIGIN
        1 cattcacttg gaggtggtac aggttctggt atgggaacat tgcttatctc gaagatccgt
       61 gaggaatatc cagatcggat tatgagctct ttttcggttg tgccatcacc taaagtatgt
      121 atatttgtgt cttaactagt ttgatttaat tttcttggtt catacctttt cgttatttag
```

```
      181 aagccatcat tttatttgct tcaaattcat gaatgaagtg aacactatct gacgagatga
      241 ttttaatctg tccctatttc cttttgaaat gcaaggttgg tttttcaggt atcagatgtt
      301 gtgttggaac cttacaatgc aacgttatca gtgcatcaat tagttgaaaa cactgatgaa
      361 actttctgca ttgataatga agctttatat gatatctgct tccgaacatt gaaattgacg
      421 aatccaactt acgg
//

Dirofilaria immitis isolate L25 beta-tubulin isotype 1 (tub-1) gene, partial cds
GenBank: MT674286.1

FASTA Graphics
Go to:
LOCUS       MT674286                 434 bp    DNA     linear   INV 20-JAN-2021
DEFINITION  Dirofilaria immitis isolate L25 beta-tubulin isotype 1 (tub-1)
            gene, partial cds.
ACCESSION   MT674286
VERSION     MT674286.1
KEYWORDS    .
SOURCE      Dirofilaria immitis (dog heartworm nematode)
  ORGANISM  Dirofilaria immitis
            Eukaryota; Metazoa; Ecdysozoa; Nematoda; Chromadorea; Rhabditida;
            Spirurina; Spiruromorpha; Filarioidea; Onchocercidae; Dirofilaria.
REFERENCE   1  (bases 1 to 434)
  AUTHORS   Intarapuk,A.
  TITLE     Direct Submission
  JOURNAL   Submitted (26-JUN-2020) Faculty of Veterinary Medicine, Mahanakorn
            University of Technology, 140 Chan Sampan rd., Nongchok, Bangkok
            10530, Thailand
COMMENT     ##Assembly-Data-START##
            Sequencing Technology :: Sanger dideoxy sequencing
            ##Assembly-Data-END##
FEATURES             Location/Qualifiers
     source          1..434
                     /organism="Dirofilaria immitis"
                     /mol_type="genomic DNA"
                     /isolate="L25"
                     /db_xref="taxon:6287"
                     /PCR_primers="fwd_name: bt93, fwd_seq:
                     ggatccggattccaactgact, rev_name: bt26, rev_seq:
                     gaattccaagtgattgagatcg"
     gene            <1..>434
                     /gene="tub-1"
     mRNA            join(<1..114,289..>434)
                     /gene="tub-1"
                     /product="beta-tubulin isotype 1"
     CDS             join(<1..114,289..>434)
                     /gene="tub-1"
                     /codon_start=1
                     /product="beta-tubulin isotype 1"
                     /protein_id="QQP23406.1"
                     /translation="HSLGGGTGSGMGTLLISKIREEYPDRIMSSFSVVPSPKVSDVVL
                     EPYNATLSVHQLVENTDETFCIDNEALYDICFRTLKLTNPTYG"
ORIGIN
        1 cattcacttg gaggtggtac aggttctggt atgggaacat tgcttatctc gaagatccgt
       61 gaggaatatc cagatcggat tatgagctct tttcggttg tgccatcacc taaagtatgt
      121 atatttgtgt cttaactagt ttgatttaat tttcttggtt cataccttt cgttatttag
      181 aagccatcat tttatttgct tcaaattcat gaatgaagtg aacactatct gacgagatga
      241 ttttaatctg tccctatttc cttttgaaat gcaaggttgg tttttcaggt atcagatgtt
      301 gtgttggaac cttacaatgc aacgttatca gtgcatcaat tagttgaaaa cactgatgaa
      361 actttctgca ttgataatga agctttatat gatatctgct tccgaacatt gaaattgacg
      421 aatccaactt acgg
//
```

```
Dirofilaria immitis isolate L26 beta-tubulin isotype 1 (tub-1) gene, partial cds
GenBank: MT674287.1

FASTA Graphics
Go to:
LOCUS       MT674287                 434 bp    DNA     linear   INV 20-JAN-2021
DEFINITION  Dirofilaria immitis isolate L26 beta-tubulin isotype 1 (tub-1)
            gene, partial cds.
ACCESSION   MT674287
VERSION     MT674287.1
KEYWORDS    .
SOURCE      Dirofilaria immitis (dog heartworm nematode)
  ORGANISM  Dirofilaria immitis
            Eukaryota; Metazoa; Ecdysozoa; Nematoda; Chromadorea; Rhabditida;
            Spirurina; Spiruromorpha; Filarioidea; Onchocercidae; Dirofilaria.
REFERENCE   1  (bases 1 to 434)
  AUTHORS   Intarapuk,A.
  TITLE     Direct Submission
  JOURNAL   Submitted (26-JUN-2020) Faculty of Veterinary Medicine, Mahanakorn
            University of Technology, 140 Chan Sampan rd., Nongchok, Bangkok
            10530, Thailand
COMMENT     ##Assembly-Data-START##
            Sequencing Technology :: Sanger dideoxy sequencing
            ##Assembly-Data-END##
FEATURES             Location/Qualifiers
     source          1..434
                     /organism="Dirofilaria immitis"
                     /mol_type="genomic DNA"
                     /isolate="L26"
                     /db_xref="taxon:6287"
                     /PCR_primers="fwd_name: bt93, fwd_seq:
                     ggatccggattccaactgact, rev_name: bt26, rev_seq:
                     gaattccaagtgattgagatcg"
     gene            <1..>434
                     /gene="tub-1"
     mRNA            join(<1..114,289..>434)
                     /gene="tub-1"
                     /product="beta-tubulin isotype 1"
     CDS             join(<1..114,289..>434)
                     /gene="tub-1"
                     /codon_start=1
                     /product="beta-tubulin isotype 1"
                     /protein_id="QQP23407.1"
                     /translation="HSLGGGTGSGMGTLLISKIREEYPDRIMSSFSVVPSPKVSDVVL
                     EPYNATLSVHQLVENTDETFCIDNEALYDICFRTLKLTNPTYG"
ORIGIN
        1 cattcacttg gaggtggtac aggttctggt atgggaacat tgcttatctc gaagatccgt
       61 gaggaatatc cagatcggat tatgagctct ttttcggttg tgccatcacc taaagtatgt
      121 atatttgtgt cttaactagt ttgatttaat tttcttggtt catacctttt cgttatttag
      181 aagccatcat tttatttgct tcaaattcat gaatgaagtg aacactatct gacgagatga
      241 ttttaatctg tccctatttc cttttgaaat gcaaggttgg tttttcaggt atcagatgtt
      301 gtgttggaac cttacaatgc aacgttatca gtgcatcaat tagttgaaaa cactgatgaa
      361 actttctgca ttgataatga agctttatat gatatctgct tccgaacatt gaaattgacg
      421 aatccaactt acgg
//

Dirofilaria immitis isolate L27 beta-tubulin isotype 1 (tub-1) gene, partial cds
GenBank: MT674288.1

FASTA Graphics
Go to:
LOCUS       MT674288                 434 bp    DNA     linear   INV 20-JAN-2021
DEFINITION  Dirofilaria immitis isolate L27 beta-tubulin isotype 1 (tub-1)
```

```
            gene, partial cds.
ACCESSION   MT674288
VERSION     MT674288.1
KEYWORDS    .
SOURCE      Dirofilaria immitis (dog heartworm nematode)
  ORGANISM  Dirofilaria immitis
            Eukaryota; Metazoa; Ecdysozoa; Nematoda; Chromadorea; Rhabditida;
            Spirurina; Spiruromorpha; Filarioidea; Onchocercidae; Dirofilaria.
REFERENCE   1  (bases 1 to 434)
  AUTHORS   Intarapuk,A.
  TITLE     Direct Submission
  JOURNAL   Submitted (26-JUN-2020) Faculty of Veterinary Medicine, Mahanakorn
            University of Technology, 140 Chan Sampan rd., Nongchok, Bangkok
            10530, Thailand
COMMENT     ##Assembly-Data-START##
            Sequencing Technology :: Sanger dideoxy sequencing
            ##Assembly-Data-END##
FEATURES             Location/Qualifiers
     source          1..434
                     /organism="Dirofilaria immitis"
                     /mol_type="genomic DNA"
                     /isolate="L27"
                     /db_xref="taxon:6287"
                     /PCR_primers="fwd_name: bt93, fwd_seq:
                     ggatccggattccaactgact, rev_name: bt26, rev_seq:
                     gaattccaagtgattgagatcg"
     gene            <1..>434
                     /gene="tub-1"
     mRNA            join(<1..114,289..>434)
                     /gene="tub-1"
                     /product="beta-tubulin isotype 1"
     CDS             join(<1..114,289..>434)
                     /gene="tub-1"
                     /codon_start=1
                     /product="beta-tubulin isotype 1"
                     /protein_id="QQP23408.1"
                     /translation="HSLGGGTGSGMGTLLISKIREEYPDRIMSSFSVVPSPKVSDVVL
                     EPYNATLSVHQLVENTDETFCIDNEALYDICFRTLKLTNPTYG"
ORIGIN
        1 cattcacttg gaggtggtac aggttctggt atgggaacat tgcttatctc gaagatccgt
       61 gaggaatatc cagatcggat tatgagctct ttttcggttg tgccatcacc taaagtatgt
      121 atatttgtgt cttaactagt ttgatttaat tttcttggtt catacctttt cgttatttag
      181 aagccatcat tttatttgct tcaaattcat gaatgaagtg aacactatct gacgagatga
      241 ttttaatctg tccctatttc cttttgaaat gcaaggttgg tttttcaggt atcagatgtt
      301 gtgttggaac cttacaatgc aacgttatca gtgcatcaat tagttgaaaa cactgatgaa
      361 actttctgca ttgataatga agctttatat gatatctgct tccgaacatt gaaattgacg
      421 aatccaactt acgg
//
```
